# Flux Balance Analysis of Cyanobacterial Metabolism: The Metabolic Network of Synechocystis sp. PCC 6803

**DOI:** 10.1371/journal.pcbi.1003081

**Published:** 2013-06-27

**Authors:** Henning Knoop, Marianne Gründel, Yvonne Zilliges, Robert Lehmann, Sabrina Hoffmann, Wolfgang Lockau, Ralf Steuer

**Affiliations:** 1 Humboldt-Universität zu Berlin, Institut für Theoretische Biologie, Berlin, Germany; 2 Humboldt-Universität zu Berlin, Institut für Biologie, Berlin, Germany; 3 CzechGlobe - Global Change Research Center, Academy of Sciences of the Czech Republic, Brno, Czech Republic; University of Illinois at Urbana-Champaign, United States of America

## Abstract

Cyanobacteria are versatile unicellular phototrophic microorganisms that are highly abundant in many environments. Owing to their capability to utilize solar energy and atmospheric carbon dioxide for growth, cyanobacteria are increasingly recognized as a prolific resource for the synthesis of valuable chemicals and various biofuels. To fully harness the metabolic capabilities of cyanobacteria necessitates an in-depth understanding of the metabolic interconversions taking place during phototrophic growth, as provided by genome-scale reconstructions of microbial organisms. Here we present an extended reconstruction and analysis of the metabolic network of the unicellular cyanobacterium *Synechocystis* sp. PCC 6803. Building upon several recent reconstructions of cyanobacterial metabolism, unclear reaction steps are experimentally validated and the functional consequences of unknown or dissenting pathway topologies are discussed. The updated model integrates novel results with respect to the cyanobacterial TCA cycle, an alleged glyoxylate shunt, and the role of photorespiration in cellular growth. Going beyond conventional flux-balance analysis, we extend the computational analysis to diurnal light/dark cycles of cyanobacterial metabolism.

## Author Summary

Phototrophic microorganisms hold great promises as a resource to generate high-value products and biofuels using only atmospheric carbon dioxide, light, and some minerals. In particular cyanobacteria, the only known prokaryotes capable of oxygen-evolving photosynthesis, have attracted recent attention as a possible chassis for the generation of third generation biofuels. Rational engineering of microorganisms is increasingly guided by large-scale reconstructions of the metabolic network of the respective organism. Such reconstructions then serve as an integrated knowledge base for all metabolic interconversions taking place during cellular growth. Here, we present and analyze such a genome-scale reconstruction for the unicellular cyanobacterium *Synechocystis* sp. PCC 6803. Taking into account several recent reconstructions, the functional consequences of unclear and dissenting pathway annotations are discussed. The model is supplemented with experimental data to validate specific reactions steps. As a specific feature of phototrophic organisms, the re-organization of metabolism in alternating diurnal light/dark cycles is studied.

## Introduction

Almost all life on Earth ultimately depends on oxygenic photosynthesis to capture solar energy and convert atmospheric carbon into organic compounds that serve as nutrients for heterotrophic organisms. Photosynthesis and the assimilation of inorganic carbon are evolutionarily old processes, with signatures RuBisCO activity, the major enzyme of carbon fixation, tracing back more than 3 billion years [1]. The presence of molecular oxygen (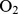) in today's atmosphere is believed to be a consequence of the appearance of cyanobacteria, ubiquitous photosynthetic microorganisms that led to the great oxygenation event, one of the major transitions in the evolution and history of life on this planet [1].

Today, cyanobacteria are the only known prokaryotes capable of oxygen-evolving photosynthesis and remain to have major impact on almost all geochemical cycles, including the global carbon cycle, global oxygen recycling and nitrogen fixation. From a metabolic perspective, cyanobacteria are highly versatile organisms and occupy diverse ecological niches where light is available. Renewed attention on cyanobacterial metabolism was triggered by the prospect to utilize their light-driven capability of 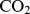 fixation for the production of high-value products [2], [3] and third generation biofuels [4]–[9]. However, to harness solar energy using cyanobacteria often requires targeted modifications of the metabolic network – a task that would greatly benefit from an in-depth understanding of metabolic interconversions taking place during phototrophic growth. A first step towards such an increased understanding is often provided by detailed and validated genome-scale reconstructions of the metabolic networks of the respective organisms. Recently, a number of metabolic reconstructions of cyanobacteria, most notably for the strain *Synechocystis* sp. PCC 6803, became available [10]–[18]. While these reconstructions differ significantly in reliability, size and scope, each led too useful insight into the metabolic organization of the model organism. In particular, analysis of different reconstructions allows us to pinpoint open questions in the representation of the metabolic network of *Synechocystis* sp. PCC 6803.

In this work, we present and interrogate an updated representation of the metabolic network of *Synechocystis* sp. PCC 6803. The updated model integrates novel results with respect to the cyanobacterial TCA cycle, an alleged glyoxylate shunt, the role of photorespiration in cellular growth, as well peculiarities of photosynthetic reactions such as light-dependent oxidative stress. In this paper, we seek to explore the implications of alternative network topologies for phototrophic flux patterns and optimal growth. Closing the iterative cycle of Systems Biology, our computational analysis is supplemented with specifically acquired experimental data to validate unclear reaction steps and growth conditions. Furthermore, we seek to improve the applicability of FBA on phototrophic conditions by implementing a full diurnal cycle, guided by the diurnal transcription of key enzymes. The manuscript is organized as follows: First, we provide a brief overview on the current status of our reconstruction of the metabolic network of the cyanobacterium *Synechocystis* sp. PCC 6803, including several computational validation steps. Subsequently, we discuss a reference condition for phototrophic growth that allows us to compare different network topologies with respect to optimal biomass yield using flux-balance analysis. Based on this reference condition, details of alternative flux solutions with respect to the TCA cycle, the glyoxylate bypass, the RuBisCO oxygenase and photorespiration are explored. In the final section, we discuss the diurnal cycles of phototrophic metabolism using a time-varying objective function.

## Results/Discussion

### The metabolic network of Synechocystis sp. PCC 6803

The starting point of our analysis is an extended and updated metabolic reconstruction of the cyanobacterium *Synechocystis* sp. PCC 6803. The reconstruction is based on a previously published network of the organism [13], and takes into account knowledge from several complementing recent reconstructions [10]–[12], [14]–[18]. As compared to our previous reconstruction of *Synechocystis* sp. PCC 6803, the network was extended to include lipid and fatty-acid metabolism, biosynthesis of peptidoglycan, chlorophylls, carotenoids, terpenoids, quinones and tocopheroles, thiamine-diphosphates, as well as the synthesis of several co-factors, vitamins and several stress related-pathways. The description of photosynthetic light reactions and transport processes was significantly improved. The reconstruction process itself followed standard procedures described in the literature [19] and is detailed in Materials and Methods. In order to avoid an inflation of network size by poorly validated reactions, we distinguish between a core network used for further computational analysis and an augmented network including all remaining annotated enzymes with putative metabolic function.

**Figure 1:**
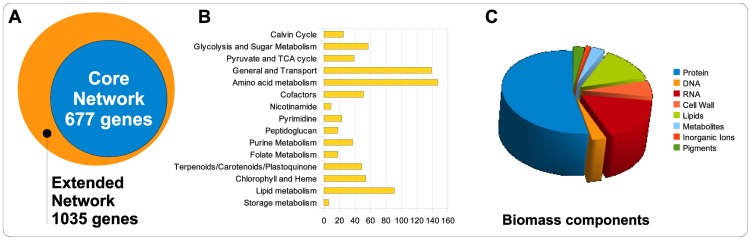
Overview of network properties. (A) We distinguish between a core network and all remaining genes annotated with enzymatic function. (B) Distribution of pathway annotations across the 760 reactions. (C) The distribution of components that define the biomass objective function (BOF), excluding storage and maintenance (ATP). Abbreviations: Terpenoids (Terp.), Carotenoids (Carot.), Plastoquinone (PQ), Tocopherol (Toco.).

The core network encompasses a connected set of all known metabolic pathways for the synthesis of main biomass and co-factors known to occur within the cyanobacterium *Synechocystis* sp. PCC 6803. During the reconstruction process, completeness of all synthesis pathways was validated with respect to biomass components, co-factors and dilution of metabolites by growth. The core reconstruction encompasses 677 genes, that encode for 495 enzymes or enzyme-complexes. The annotated enzymes give rise to 759 metabolic reactions among 601 metabolites. In addition, the core reconstruction contains 6 spontaneous interconversions and 61 transport reactions, including diffusion. The reaction network distinguishes between six cellular compartments, the cytosol, the thylakoid membrane, the thylakoid lumen, the plasma membrane, the periplasmic space, carboxysomes, as well as the extracellular space. Several key properties of the network are summarized in Figure 1. The reconstruction is provided as an annotated SBML file (System Biology Markup Language, [20]) and as an Excel sheet, Supplementary Dataset S1 and Supplementary Table S1, respectively. Annotated enzymes that are not part of the core network are provided as Supplementary Table S2. To enhance usability of the reconstructed network, a detailed graphical overview of the metabolic network was prepared and is provided as Figure S1.

### Flux-balance analysis

Large scale metabolic reconstructions offer the possibility to investigate physiological properties of the respective organism using constraint-based analysis [21]. While flux-balance analysis was already employed with great success for a variety of heterotrophic unicellular organisms, the application on phototrophic growth is still in its infancy [22]. In particular, phototrophic growth gives rise to additional computational and conceptual challenges, such as diurnal patterns of light availability. However, before such more complex scenarios can be considered, we need to establish a reference solution for phototrophic growth under constant light. Following the practice of conventional FBA, we assume that intracellular fluxes are organized such that they realize a given cellular objective function, namely maximal biomass yield, for given constraints and exchange fluxes. The biomass objective function (BOF) was adapted and modified from Nogales et al. [17] and consists of proteins, DNA, RNA, cell wall components, lipids, soluble metabolites, inorganic ions and pigments. Growth-dependent ATP utilization is included to account for energy requirement of protein synthesis and growth.

In addition to cellular growth, the reference solution must accommodate additional processes known to affect cyanobacterial metabolism. We assume a basal growth-independent ATP utlilization for cellular maintenance. Likewise, cyanobacteria are assumed to exhibit residual respiratory activity also during light [23], [24]. A number of further processes are directly dependent on light input, such as creation of reactive oxygen species (ROS), photoinhibition and photodamage. Scavenging of ROS may result in increased demand of NADPH, whereas photodamage results in high growth-independent turnover of cellular components, most notably the D1 protein of photosystem II [25]. Recently, also the existence of a Mehler-like reaction, differing from its counterpart in higher plants in producing no reactive oxygen species, has been demonstrated [26], [27]. Unfortunately, quantitative data on these processes are scarce and their interdependencies with growth are only incompletely understood.

In order to constrain and parametrize the flux-balance solution under constant light, we assume an average doubling time of 24 h under constant illumination, corresponding to an average growth rate of 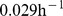. Carbon uptake is not constrained and taken up only as bicarbonate (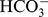). We only consider net exchange fluxes and do not explicitely account for cycling of inorganic carbon [28]. In addition to growth-related ATP utilization, a basal maintenance of 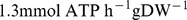 is included. To account for basal respiratory activity in the light, we assume nonzero activity of the terminal oxidase, as well as of the Mehler-like reaction that converts NADPH and 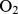 to 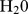 and NADP. Both processes are assumed to take up 10% of 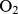 evolution of photosystem II, respectively. However, we note that estimates of the respective activities strongly vary in the literature and seem to be highly dependent on the specific growth conditions. For the activity of the terminal oxidase, we follow the values of Helman et *al.* [29], who assessed the extent of electron flow via cytochrome oxidase in the light and concluded that 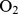 consumption by respiratory activity in the light was about 6% that of 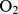 production. The Mehler-like reaction was recently studied by Allahverdiyeva et *al.* [27], who report that under air level 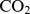 conditions approximately 20% of electrons originating from water splitting are targeted to 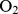 – mainly due to the Mehler-like reactions. We note that the precise values used here may need revision in future studies, but do not qualitatively affect the properties of the optimized flux solution. To account for oxidative stress, superoxide (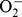) is created in photosystem II (PSII), and at photosystem I (PSI) by the plant-type Mehler reaction. Both processes are assumed to be very low and are assumed to correspond only to 0.5% of the respective electron flow. Nitrogen is taken up as nitrate (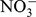) using an ABC transporter. In the following, unless stated otherwise, light is considered to be the growth limiting factor. Other nutrients, including nitrogen, phosphorus, and sulfur, are not considered limiting. We do not consider uptake of complex molecules, such as glucose or amino acids.

### Metabolic flux during phototrophic growth

**Figure 2:**
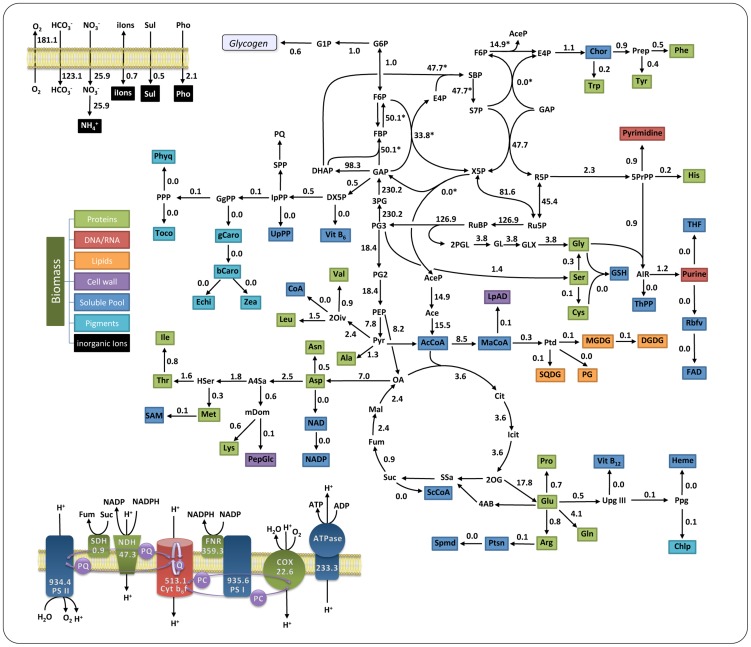
A flux map for phototrophic growth under constant light. The flux distribution was optimized for maximal biomass yield. Shown are flux values in units of 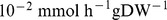. Flux values marked with an asterisk indicate non-unique values. The solution is characterized by a large flux through the CBB cycle and non-cyclic operation of the TCA cycle. Aerobic respiration and oxygenic photosynthesis share common components in the electron transport chain. During phototrophic growth, electrons originate from water splitting in PS II and are transferred to NADPH via PS I and FNR. Abbreviations are defined in the Materials and Methods.

Given the constraints and conditions defined above, a solution for the flux-optimization problem was obtained using the COBRA toolbox [30] and verified using FASIMU [31], both giving identical results. The reference solution under constant light is not unique. A graphical overview is given in Figure 2. Overall, the solution is in good agreement with previous studies [16], [17]. As expected, autotrophic growth is based on assimilation of carbon dioxide by the ribulose-1,5-bisphosphate carboxylase/oxygenase (RuBisCO, EC 4.1.1.39). RuBisCO converts one molecule of ribulose-1,5-bisphosphate (RuBP) and 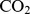 to two molecules of glycerate-3-phosphate (PG3). To ensure sustained growth, PG3 is then utilized to regenerate RuBP via the Calvin-Benson-Bassham (CBB) cycle, resulting in a surplus of one molecule of PG3 for each 6 molecules of PG3 generated by the cycle. Energy (ATP) and reducing power (NADPH) are provided by the photosynthetic light reactions. Beyond the CBB cycle, flux towards biomass synthesis drops considerably in terms of absolute magnitude. During phototrophic growth, flux through the tricarboxylic acid (TCA) cycle is non-cyclic and acts as a hinge to distribute metabolic precursors for growth. Within the TCA cycle fumarate that originates as a by-product of purine synthesis is re-channeled into metabolism. In addition to maximizing the biomass objective function, the flux solution for phototrophic growth also incorporates synthesis of storage compounds that are then re-utlized during periods of darkness. For simplicity, within our computational analysis, we consider glycogen as the only storage compound. The corresponding flux is 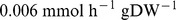. We observe no qualitative changes in flux distribution for varying light intensity, as long as light remains the growth limiting factor.

To discuss the validity of the computational flux-optimization, a comparison with experimentally obtained flux values is crucial. To this end, Young et al. [32] recently presented a photoautotrophic flux map based on dynamic isotope labeling measurements. The experimental flux distribution is in good agreement with results obtained with FBA, notwithstanding several interesting differences. When optimizing for maximal biomass yield, the computational solution consistently assigns non-zero flux through the phosphoketolase (PHK, EC 4.1.2.22) that converts either xylose 5-phosphate (X5P) to acetyl phosphate (AceP) and glyceraldehyde 3-phosphate (GAP) or fructose 6-phosphate (F6P) to acetyl phosphate and erythrose 4-phosphate (E4P). The phosphoketolase therefore effectively acts as a shortcut from the CBB cycle to acetyl coenzyme A (acetyl-CoA) and as a bypass of the 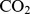 releasing reaction catalyzed by the pyruvate dehydrogenase complex (PDH). Interestingly, the phosphoketolase pathway was previously discussed as an innovative solution for pentose catabolism in *Saccharomyces cerevisiae* [33] and L-glutamate production in *Corynebacterium glutamicum* [34]. The presence of the phosphoketolase also has a significant influence on the PGK/PGM branchpoint that diverts flux from the CBB cycle. With non-zero flux through PHK, the PGK/PGM ratio is approximately 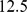, whereas in the absence of the PHK, the ratio is 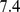, in agreement with previous studies [13], [17]. The value determined experimentally by Young et al. [32] is approximately 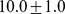, allowing no definite conclusions on the validity of either solution.

A further discrepancy between the two flux maps is the role of the malic enzyme (ME) during phototrophic growth. In accordance with earlier studies on heterotrophic growth [35], Young et al. [32] report a non-zero flux through ME, suggesting that the enzyme might be involved in concentrating intracellular 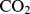 analogous to its role in C4 plants. However, since such a cycle dissipates energy, FBA will not select for a non-zero flux in the absence of further constraints. Rather surprisingly, the computational study of Nogales et al. [17] nonetheless report such a non-zero flux through the ME. However, this study considered carbon limited growth in the excess of light – resulting in various energy dissipating cycles. Indeed, flux variability analysis of the model of Nogales et al. [17] shows that absence of flux through the ME reaction is likewise compatible with optimal growth.

Finally, the experimental study of Young et al. [32] reveals an unexpected residual flux through the oxidative pentose (OPP) pathway. As during phototrophic growth, NADPH is excessively produced by the light-driven electron transport chain (ETC), the flux through OPP pathway fulfills no obvious cellular requirements and only results in small but significant loss of fixed carbon. As argued by Young et al. [32], the detected flux may therefore represent an incomplete suppression of OPP pathway during night/day transitions. Again, such a suboptimal flux state is not recovered by FBA in the absence of additional constraints. However, it demonstrates the utility of FBA to identify suboptimal solutions and the necessity to also consider sub-optimal states in network analysis. Indeed, it has been shown previously that cellular metabolism can maintain a ‘standby mode’ in anticipation of changing environmental conditions at the expense of optimal growth [36], [37]. This trade-off between flexibility and efficiency requires the investment of additional resources and is likely to affect diurnal growth of photosynthetic organisms.

### Metabolic flux during periods of darkness

In addition to phototrophic growth, *Synechocystis* sp. PCC 6803 has to survive extended periods of darkness, usually relying on endogenous storage compounds that are accumulated when light is available. Unfortunately, data on experimental flux patterns under prolonged darkness are scarce. To nonetheless approximate metabolic flux under periods of darkness, we assume that, unlike for some diazotrophic cyanobacteria, dark metabolism in *Synechocystis* sp. PCC 6803 is dominated by a low level of cellular maintenance and hence utilization of ATP. Growth is assumed to be minimal, which is in good agreement with experimental observations. As a constraint for the flux-optimization problem, we therefore allow for a maximal rate of glycogen consumption that only slightly exceeds the requirements for respiratory metabolism and the demand of ATP. In addition to ATP utilization, we assume residual growth, or, equivalently, a residual cellular turnover that is likewise approximated by the biomass objective function [13]. The dark optimization problem therefore seeks to maximize the BOF under conditions of limited glycogen utilization that is only slightly above the requirement for cellular maintenance. We note that, while glycogen is a major respiratory substrate during periods of darkness, *Synechocystis* sp. PCC 6803 is likely to utilize other substrates as well. As shown recently, mutants impaired in glycogen synthesis have strongly reduced viability in dark-night cycles – however the reduction in viability is not strong enough to confirm glycogen as only storage compound [38]. In the following, the contributions of alternative storage compounds are not considered.

The resulting optimal flux patterns shows considerable variability in dependence of detailed assumptions about enzyme specificity and directionality. When the annotated transhydrogenase reaction (*slr1239* and *slr1434*, EC 1.6.1.2) is assumed to be active and allowed to carry reversible flux, redox potential (NADH) for respiration is generated via cyclic flux through the TCA cycle and subsequently converted into NADPH. NADPH is mainly fed into the NADPH dehydrogenase complexes (NDH-1). NDH-1 was reported to be specific for NADPH [23]. A different flux pattern emerges, if the transhydrogenase is assumed to be either absent or only unidirectionally converting NADPH into NADH. In this case, the computational solution suggests that redox potential for respiration (NADPH) is predominantly generated by the OPP pathway, with no cyclic flux through the TCA cycle. Utilization of the OPP would be in good agreement with the observation that during heterotrophic growth, a large fraction of the consumed glucose was reported to be oxidized via the OPP pathway [35]. However, if we allow the respiratory complex NDH-1 to utilize both, NADH and NADPH, as substrates then again cyclic flux through the TCA is predicted by the model. The use of the cyclic TCA cycle is also supported by the observation that the succinate dehydrogenase reaction is the main respiratory electron transfer pathway into the PQ pool [23]. The actual flux pattern during periods of darkness cannot be resolved based on the presently available data. We favor a scenario with cyclic flux through the TCA cycle. The estimated flux distributions for dark metabolism are provided as Supplemental Table S3.

### RuBisCO oxygenase and photorespiration

Continuing with the analysis under conditions of constant illumination, we seek to discuss specific features of phototrophic metabolism in more detail. In particular, under current atmospheric conditions, photosynthetic productivity is significantly impaired by the fact that RuBisCO exhibits a nonzero affinity for molecular oxygen, instead of 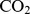, as an alternative substrate. Mostly regarded as an evolutionary relic of the 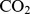-rich atmosphere in which RuBisCO first evolved, photorespiration is a seemingly wasteful process and effectively withdraws carbon from the CBB cycle. However, recently, also alternative hypotheses have emerged that suggest an essential role for photorespiration in energy-dissipation [39] and other processes [40]. As a rather surprising result, our previous reconstruction has shown that optimization for maximal biomass yield gives rise to a nonzero rate of photorespiration, roughly matching reported experimental values [13]. The reason for this apparent non-optimal route was the absence of stoichiometrically more efficient pathways for the synthesis of the amino acids serine, glycine and cysteine. In particular, as yet, the enzymes phosphoserine transaminase (EC 2.6.1.52) and phosphoserine phosphatase (EC 3.1.3.3) have no known homologues in the genome of *Synechocystis* sp. PCC 6803. While a candidate for the latter was recently suggested [41], and possible candidates for the former are available [M. Hagemann, personal communication], the pathway must currently still be regarded as incomplete in *Synechocystis* sp. PCC 6803. In the absence of an annotated phosphoserine pathway in the metabolic reconstruction, glycine is produced from glyoxylate as a by-product of photorespiration.

**Figure 3:**
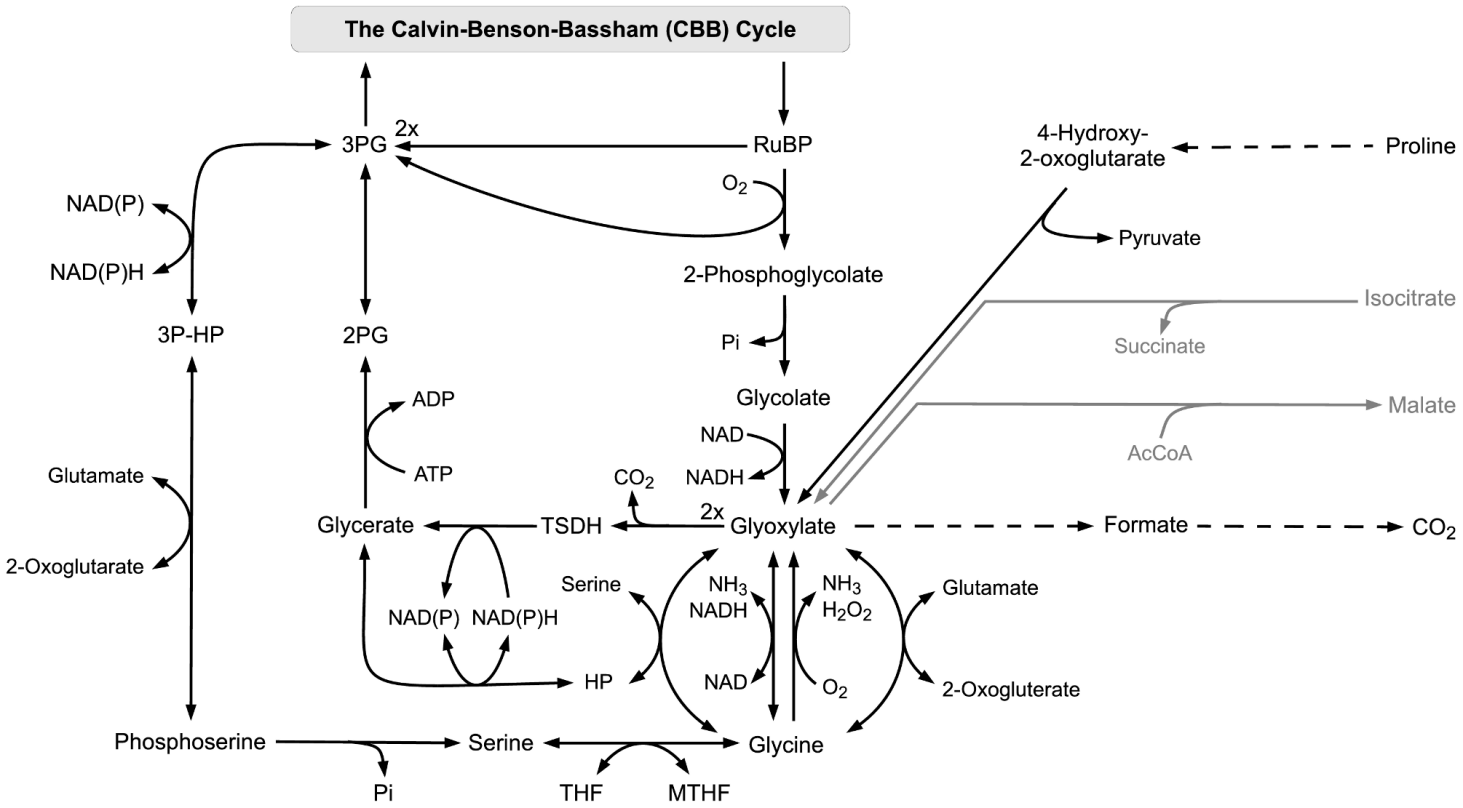
RuBisCO oxygenase and photorespiration.

The situation is similar within the current reconstruction. Optimization with respect to biomass yield again results in a nonzero rate of photorespiration. We therefore investigated three scenarios related to the possible production of the amino acids glycine and serine. The first scenario assumes that the current annotation is not incomplete and the enzymatic activity of a phosphoserine transaminase and phosphoserine phosphatase are indeed absent in *Synechocystis* sp. PCC 6803. In this case, glycine, serine, and cysteine are synthesized from glyoxylate that itself is a product of glycolate and hence 2-phosphoglycolate (2PG), the product of photorespiration. A non-zero rate of photorespiration therefore emerges as a result of the flux optimization problem. See Figure 3 for a pathway map. Quantitatively, the optimal rate of photorespiration is approximately 5%, well within current estimates of photorespiration [42]. However, we note that Young et al. [32] observed a considerably lower rate of photorespiration in similar experimental conditions.

Reverting to a simulation of dark metabolism, as defined above, photorespiratory flux is no longer part of the optimal solution. Instead, the residual demand for glycine, serine and cysteine is met by degradation of proline, resulting in a slightly higher yield than photorespiration under non-phototrophic conditions. As argued previously [13], this switch also shows that stoichiometric efficiency is not a property of an isolated pathway, but must be considered in the context of the flux solution as a whole. We note that this solution is different from the computational results of Nogales et al. [17], who suggest that RuBisCO oxygenase, but not carboxylase, is active under heterotrophic conditions.

As our second scenario, we assumed that as yet unidentified genes encode for a phosphoserine transaminase and phosphoserine phosphatase. When the respective enzymatic steps are introduced into the reconstruction, photorespiration ceases under phototrophic conditions and the RuBisCO oxygenase is no longer part of the optimized solution. Instead serine, and subsequently glycine and cysteine, are produced by the newly introduced phosphoserine pathway. This solution is also in agreement with the results of Young et al. [32] and Huege et al. [42] who both observed that 13C-enrichment of serine was substantially higher than glycine during transient labeling.

Within the third scenario, taking into account that photorespiration is presumably an inevitable process under current atmospheric conditions, we investigated the optimal flux distribution in the presence of the phosphoserine pathway while simultaneously forcing a non-zero flux of the RuBisCO oxygenase. Specifically, the flux through RuBisCO oxygenase is constrained with a lower bound of 3% of the carboxylase flux. In this case, flux variability analysis shows that several equivalent flux solutions exists. The photorespiratory intermediate glyoxylate may either be used for the synthesis of glycine, or, stoichiometrically equivalent in terms of biomass yield, may be recycled into the CBB cycle via glycerate. In the latter case, serine is synthesized via the phosphoserine pathway. The corresponding pathway maps are shown in Figure 3. Overall, the question of the possible activity of the phosphoserine pathway still represents an evolutionary conundrum. Given that photorespiration is considered an inevitable side process under current atmospheric conditions, it seems advantageous to use its products in the stoichiometrically most efficient way. In particular, there seems little incentive to establish or maintain an alternative pathway that results in a stoichiometrically identical yield. Interestingly, the absence of such an alternative pathway would make cellular metabolism dependent on a wasteful side product, which in turn might impede further optimization of the RuBisCO reaction: an evolutionary deadlock. Nonetheless, there is also indirect evidence for the phosphoserine pathway, in particular from transient labeling experiments [32]. Furthermore, one step of the phosphoserine pathway, a 3-phosphoglycerate dehydrogenase (EC 1.1.1.95), is annotated in the genome of *Synechocystis* sp. PCC 6803. The gene was recognized as a hydroxypyruvate reductase (EC 1.1.1.81) in the work of Eisenhut et al. [43]. However, recent evidence indicates that the gene indeed encodes a 3-phosphoglycerate dehydrogenase and an alternative candidate for the hydroxypyruvate reductase has been identified [Martin Hagemann, personal communication].

As a preliminary conclusion, we therefore favor a scenario where the phosphoserine pathway is present, albeit encoded with as yet unidentified genes. In the following, all simulations correspond to the third scenario studied above, with RuBisCO oxygenase activity forced as 3% of it carboxylase activity. The phosphoserine pathway then functions as an auxiliary supply of serine that allows to cope with varying levels of photorespiration.

### The cyanobacterial glyoxylate shunt

Several recent reconstructions of the metabolic network of *Synechocystis* sp. PCC 6803 include the metabolic reactions isocitrate lyase (ICL, EC 4.1.3.1) and malate synthase (EC 2.3.3.9), encoding a bacterial glyoxylate shunt [10], [12], [14]. Isocitrate lyase, the first enzyme of the glyoxylate shunt, splits isocitrate to succinate and glyoxylate. While the corresponding genes are not annotated within the genome, the decision to include the glyoxylate shunt was partly motivated by reports that the respective enzymatic activities have been detected experimentally [35], [44], [45]. Also, genes for a functioning glyoxylate shunt were recently identified in the genome of Cyanothece strains [46], albeit with no homologues in *Synechocystis* sp. PCC 6803. However, as argued previously [13], the experimental reports are not conclusive. To resolve the discrepancy and to test the functional implications of a glyoxylate shunt in phototrophic metabolism, we therefore performed experimental validation of the enzymatic steps and investigated different scenarios using constraint optimization. When the isocitrate lyase is introduced into the current reconstruction, the enzymatic step is indeed utilized within the optimized flux distribution. In this case, isocitrate lyase is used to synthesize glyoxylate, providing a precursor for the amino acids glycine, serine, and cysteine. Correspondingly, under these conditions, the photorespiratory flux within the optimized computational flux solution is zero. However, if the phospho-serine pathway is assumed to be present, no flux through either the full glyoxylate shunt or the isocitrate lyase is obtained. Likewise, flux through the isocitrate lyase is obtained for dark metabolism, providing glyoxylate for cellular turnover. It is noted that for the reconstruction of Shastri and Morgan [10], the glyoxylate shunt was introduced to close the otherwise incomplete cyanobacterial TCA cycle. Indeed, under some conditions, the isocitrate lyase has a predicted non-zero flux in dark metabolism, beyond the synthesis of glyoxylate, that is discussed in more detail below.

To experimentally resolve the possibility of enzymatic activity of the isocitrate lyase in *Synechocystis* sp. PCC 6803, we applied a refined methodology to determinate isocitrate lyase activity in cell-free extracts of *Synechocystis* sp. PCC 6803. The method was adopted from Dixon and Kornberg [47], a standard procedure for detection and quantification of glyoxylate in soluble extracts. Glyoxylate reacts with phenylhydrazine to a phenylhydrazone that can be measured by absorption at 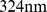. However, other metabolites with reactive keto or aldehyde groups lead to the same reaction. Particular attention must therefore be paid to 2-oxoglutarate, likewise a product of isocitrate, resulting from decarboxylation catalyzed by the isocitrate dehydrogenase in presence of the co-substrate NADP.

**Figure 4:**
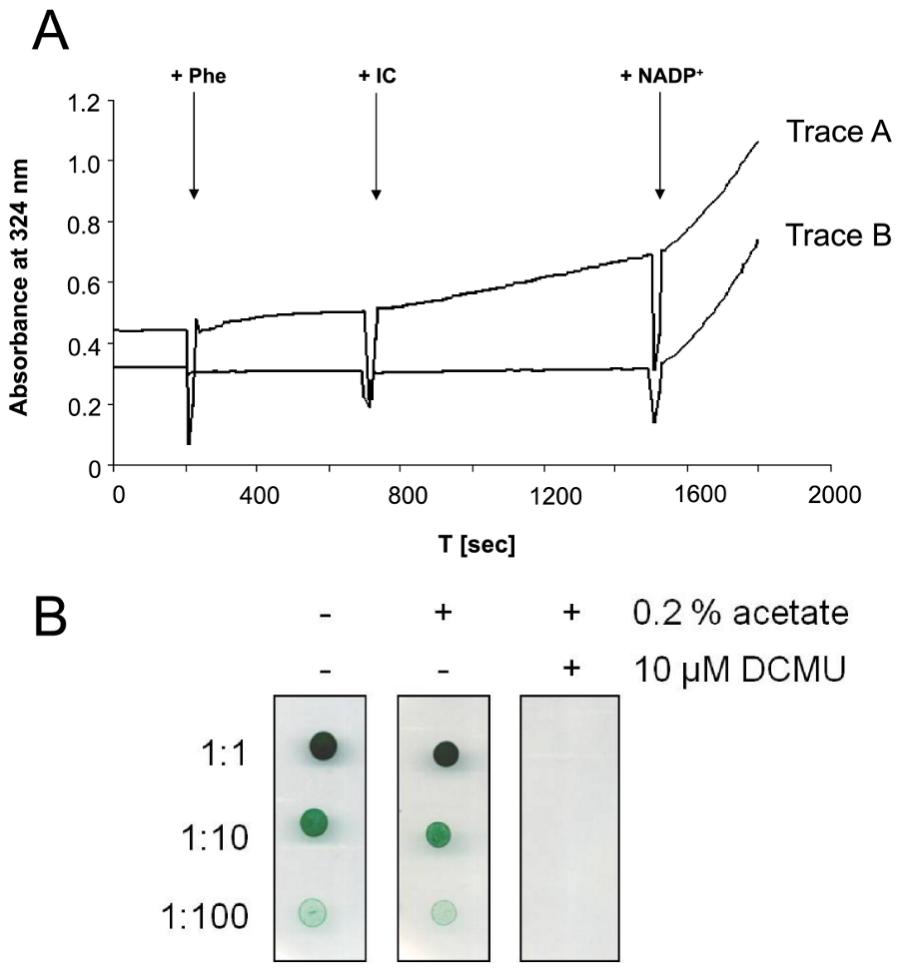
Experimental validation of the glyoxylate shunt. (A) Enzymatic test to determine isocitrate lyase activity in cell-free extract of *Synechocystis* PCC 6803. Phenylhydrazon formation were measured as increase of A324 nm with time. Trace A - crude cell extract of *Synechocystis* cells, Trace B - crude cell extract passed over a PD-10 gel filtration column both corresponding to a protein concentration of 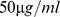 reaction volume. Arrows mark the stepwise addition of phenylhydrazin (Phe, 5 mM), isocitrate (IC, 1 mM) and NADP (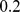 mM), the latter as a control for isocitrate dehydrogenase activity. Increase in A324 nm in Trace A after adding of phenylhydrazin results from phenylhydrazine reactive metabolites, further increase after addition of IC results from internal NADP as a co-substrate of isocitrate dehydrogenase within the crude extract. Both traces show the same trend after addition of NADP, corresponding to the same isocitrate dehydrogenase activity. (B) The capability for utilization of acetate as sole carbon source was tested in spot assays in the presence of the photosystem II inhibitor DCMU (photoheterotrophic growth). Respective controls were conducted without the addition of DCMU (putative photomixotrophic growth) and without both sodium acetate and DCMU, respectively (photoautotrophic growth) to BG11 agar. 1∶1, 1.10 and 1∶100 represent dilution factors of the stock cell suspension of *Synechocystis* sp. PCC 6803 that contains 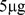 chlorophyll a per ml cell suspension.

Measurements were therefore performed with crude extracts of *Synechocystis* cells, passed over a PD-10 gel filtration column to remove small reactive metabolites and NADP. There is no significant isocitrate lyase activity detectable in filtered crude extracts of *Synechocystis* sp. PCC 6803 (Figure 4, Trace B). Positive control for ICL activity is provided in Supplemental Text S1 and Figure S4. If the filtration step was omitted, a small increase in A324 nm was measured even in the absence of isocitrate by reactive metabolites in the crude extract. After addition of isocitrate a much higher increase in A324 nm is present (Figure 4, Trace A), similar to measurements of other authors [44], [45]. These results demonstrate that the measured increase of A324 nm in crude extracts that were not passed over the gel filtration column primarily results from the formation of 2-oxoglutarate, analyzed by an alternative enzymatic test for 2-oxoglutarate quantification (not shown). The residual activity is due to the presence of small amounts of NADP in the unfiltered crude cell extract that, after reduction to NADPH, can be reoxidized by unspecific oxydases resulting in the cyclic formation of NADP, the co-substrate of isocitrate-dehydrogenase. Therefore, we conclude that the presumed isocitrate lyase activity observed by other authors is most likely the result of the isocitrate dehydrogenase reaction. Further evidence for the absence of a glyoxylate shunt is provided by the fact that *Synechocystis* sp. PCC 6803 lacks the capability to utilize acetate as sole carbon source in the presence of the photosystem II inhibitor DCMU (photoheterotrophic growth). Model simulations clearly show that in such a case, in the presence of the glyoxylate, photoheterotrophic growth is possible. Experimental results are shown in Figure 4B.

### The cyanobacterial TCA cycle

**Figure 5:**
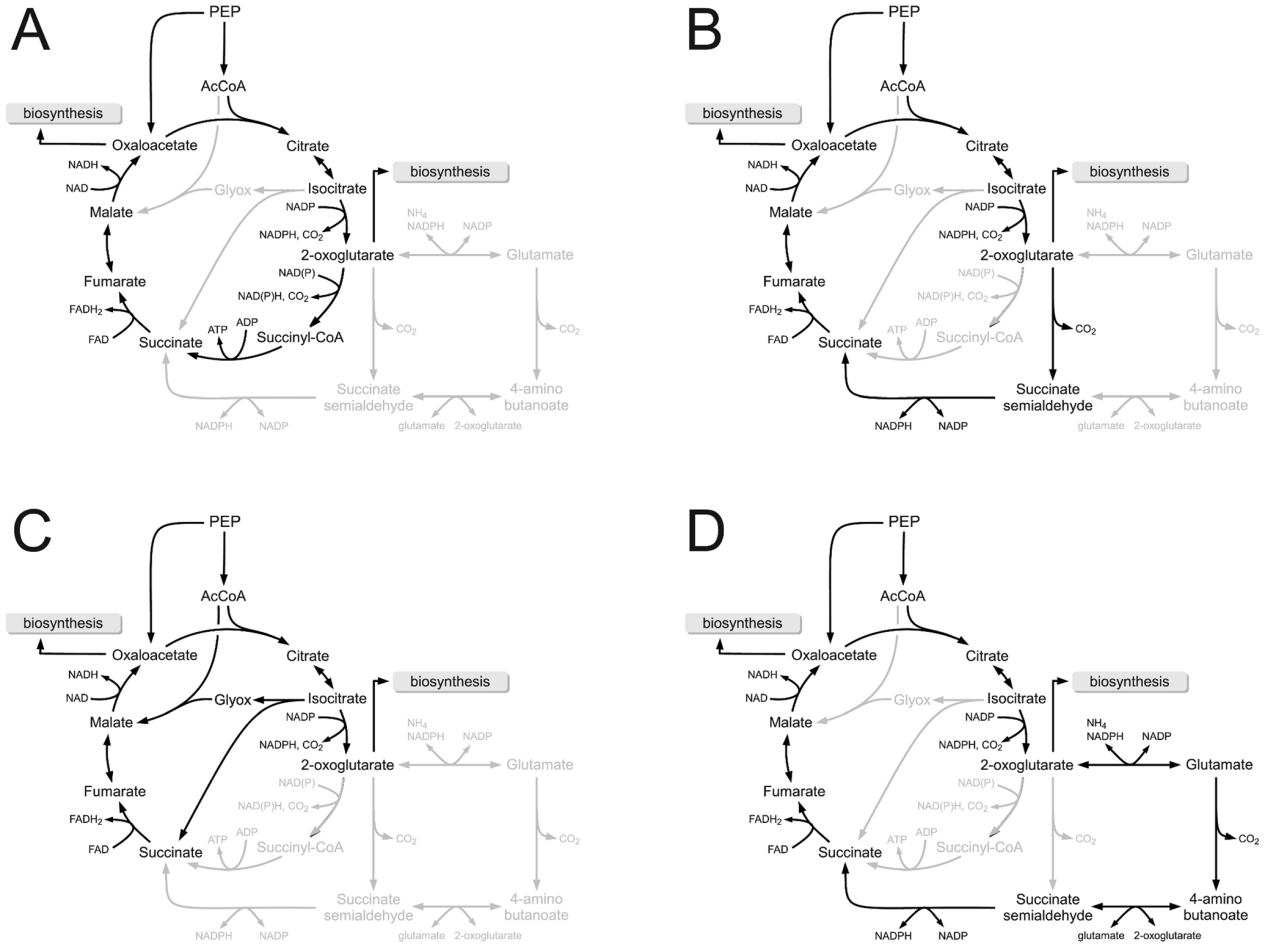
Alternative scenarios of flux through the TCA cycle. (A) The conventional closed bacterial TCA cycle via the OGDH complex. (B) A bypass recently identified by Zhang and Bryant [49]. (C ) The glyoxylate shunt as a bypass of the TCA cycle, as utilized in the reconstruction of Shastri and Morgan [10]. (D) The GABA shunt as a bypass of the TCA cycle, as utilized in the reconstruction of Knoop et al. [13]. In terms of stoichiometric yield, the conventional cycle is most effective.

As one of the most iconic pathways in central metabolism, the TCA cycle has a dual role of oxidizing respiratory substrates for ATP synthesis and providing precursor metabolites, such as oxaloacetate and 2-oxoglutarate, for biosynthesis [48]. Until recently, it was widely assumed that cyanobacteria have an incomplete TCA cycle and lack the genes encoding for the 2-oxoglutarate dehydrogenase (OGDH) complex. Correspondingly, almost all published reconstructions to date incorporate only an incomplete TCA cycle and rely on auxiliary reactions to allow for cyclic flux. For example, the analysis of Shastri and Morgan [10] assumed the presence of a glyoxylate shunt to close the cycle. Within the reconstruction of Knoop et al. [13] flux is channeled through the GABA shunt, constituting a bypass from 2-oxoglutarate, via glutamate, 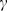-aminobutyrate (GABA) and succinate semialdehyde, to succinate. However, recently, the misconception about the incompleteness of the cyanobacterial TCA cycle was corrected [49]. Many cyanobacteria, including *Synechocystis* sp. PCC 6803, have genes encoding for two enzymes that replace the lacking OGDH complex: A 2-oxoglutarate decarboxylase (*sll1981*, EC 4.1.1.71) and a succinate semialdehyde dehydrogenase (*slr0370*, EC 1.2.1.16). Together these two enzymes constitute a shortcut that closes the incomplete TCA cycle. See Figure 5 for a corresponding pathway map.

The recent discovery of a closed cyanobacterial TCA cycle calls to reconsider the role of the cycle under different growth conditions. As outlined above, the photoautotrophic flux map obtained from FBA shows that during phototrophic growth the TCA cycle carries non-cyclic flux. In this case, the TCA cycle mainly provides oxaloacetate and 2-oxoglutarate for growth and incorporates fumarate originating in purine synthesis back into metabolism. According to the computational flux map, the two newly discovered enzymes carry no flux – consistent with the autotrophic lifestyle and highlighting the fact that the conventional closed TCA cycle is only one way how that flux through the component reactions can be organized [48], [50]. However, absence of flux through the TCA shortcut during phototrophic growth is not fully concordant with the reported experimental observation that, at least for *Synechococcus* sp. PCC 7002, mutants lacking the OGDH complex and SSADH grow at a slower rate than the wildtype also during constant illumination, as well as in light-dark cycles [49].

The predicted optimal flux pattern changes in periods of darkness. Apart from special conditions, respiratory metabolism in the dark phase typically requires cyclic flux through the TCA cycle to drive ATP synthesis. To evaluate the role of cyclic flux during periods of darkness, we distinguish between four putative scenarios to close the cyanobacterial TCA cycle: (i) a conventional bacterial TCA cycle, involving an OGDH complex that is not annotated in cyanobacteria; (ii) the actual cyanobacterial TCA cycle using a 2-oxoglutarate decarboxylase and a succinic semialdehyde dehydrogenase; (iii) an incomplete TCA cycle that is closed by the GABA shunt to establish cyclic flux through the cycle; as well as (iv) a glyoxylate shunt, via isocitrate lyase activity, to establish cyclic flux through the cycle.

Within our previous reconstruction [13], flux through the GABA shunt was used during respiratory metabolism. The respective sequence of reactions, shown in Figure 5D, is stoichometrically identical to the newly discovered shortcut of Zhang and Bryant [49], shown in Figure 5B.

Therefore, in the context of FBA, both cycles result in identical yield. Interestingly, this yield is below the yield of the conventional cycle using a OGDH complex, therefore representing a seemingly sub-optimal solution for respiratory metabolism. To solve this evolutionary conundrum, Nogales et al. [17] argue that the GABA shunt may nonetheless be an evolutionary favorable solution – based on the finding that the flux forced through the GABA shunt during phototrophic growth results in no reduction of growth, as compared to flux forced through the OGDH complex. However, this difference cannot be recovered using the TCA bypass identified by Zhang and Bryant [49]. An explanation of this discrepancy is provided in Materials and Methods. Rather, the explanation for the stoichiometric inefficiency of the cyanobacterial TCA bypass using a 2-oxoglutarate decarboxylase and a succinic semialdehyde dehydrogenase, instead of the conventional OGDH complex, might be the difference in protein synthesis requirements of both pathways. The OGDH complex is a highly sophisticated multiprotein machine, analogous to the pyruvate dehydrogenase (PDHC) complex, and is encoded by three subunits. In *E. coli*, the complex consists of a 24-mer core of its E2 component, encoded by the gene *sucB*, with an as yet unclear stoichiometry of its two other components [51], [52]. In contrast, both, the bypass of Zhang and Bryant [49], as well as the GABA shunt are encoded by comparatively simple enzymes. An overview of amino acid requirements is provided in Supplemental Table S5. Given the complexity of the OGDH complex and the relative unimportance of cyclic flux through the TCA cycle for phototrophic growth, such a difference in enzyme investment may result in a trade-off between enzymatic efficiency and enzyme synthesis costs. Indeed, it is increasingly recognized that maximization of molar yield is not necessarily a universal principle of metabolism [53], [54]. Interestingly, also the isocitrate lyase, if inserted into the model, results in a slightly higher biomass yield during dark metabolism. In this case, succinate is utilized by the succinate dehydrogenase (SDH) to close the flux through the TCA cycle.

### Temporal coordination of phototrophic metabolism

In most natural habitats cyanobacterial metabolism is subject to a diurnal cycle of light availability, resulting in significant change and re-organization within the metabolic network. Correspondingly, cyanobacteria are the only known prokaryotes with an endogenous circadian clock that acts as an intracellular zeitgeber [55]. Considerable effort has been invested to elucidate the cyclic behavior of cyanobacterial metabolism using high-throughput data [22], [56]–[60]. However, all current large-scale reconstructions exclusively focus on heterotrophic growth or phototrophic growth under constant illumination.

Here, we seek to augment the picture by an analysis of the temporal coordination of cyanobacterial metabolism, by simulating the diurnal cycle of phototrophic metabolism. To incorporate circadian changes into a large-scale model of metabolism is not trivial. In general two approaches are available: Following a bottom-up approach, large-scale data on transcript or protein expression may be used to constrain the availability of certain enzymatic interconversions. However, transcript or protein abundance must not necessarily correspond to metabolic flux and often contradicting expression values for single pathways or isoenzymes are observed. Indeed, a recent analysis of paired mRNA-protein abundance in light-dark synchronized cultures of the cyanobacterium *Prochlorococcus* MED4 showed only poor correlation between mRNA and protein abundance [60]. Also, only small changes in relative enzyme abundance over the entire time-course were observed [60]. We therefore conjecture that a straightforward integration of transcriptomic data to constrain metabolic flux, as already applied for heterotrophic bacteria [61], is not a suitable strategy to describe the periodic diurnal cycle of cyanobacterial metabolism.

Instead, we follow a top-down approach, such that the cellular objectives are defined as a function of time and change in accordance with light availability. Specifically, we use a recently obtained dataset on cyclic transcript behavior in *Synechocystis* sp. PCC 6803 [79] to obtain insight into the temporal coordination of cyanobacterial metabolism. The resulting expression patterns of metabolic enzymes, provided as Figure S2, give important references to constrain the temporal coordination of phototrophic growth. In particular, unlike for some diazotrophic strains [56], the overwhelming majority of oscillatory genes peak during day, indicating a strongly reduced expression activity during the night. Among those transcripts whose expression is highest during night, most are associated with transport processes and, to a lesser extend, TCA cycle activity – in particular the transcripts corresponding to the TCA bypass identified by Zhang and Bryant [49]. Indeed, given that many transport processes relate to the uptake of growth-limiting micronutrients, such as iron or manganese, there is little reason to assume that transport ceases during night. We then have to augment the view from expression data with physiological data obtained for *Synechocystis* or other cyanobacterial strains. For example, for *Cyanothece* sp. ATCC 51142 measurements of biomass and chlorophyll concentration, both by optical density proxy, indicate that chlorophyll concentration rises sharply from early morning to well before noon, and remains constant afterwards [57]. In contrast, biomass, does only significantly increase after the end of chlorophyll accumulation, corresponding to increase in storage compounds [57]. Furthermore, for *Synechococcus* PCC 7942 it was reported that, while cells are dividing rhythmically, DNA synthesis proceeds at a constant rate, effectively uncoupling DNA synthesis and cell division [62].

**Figure 6:**
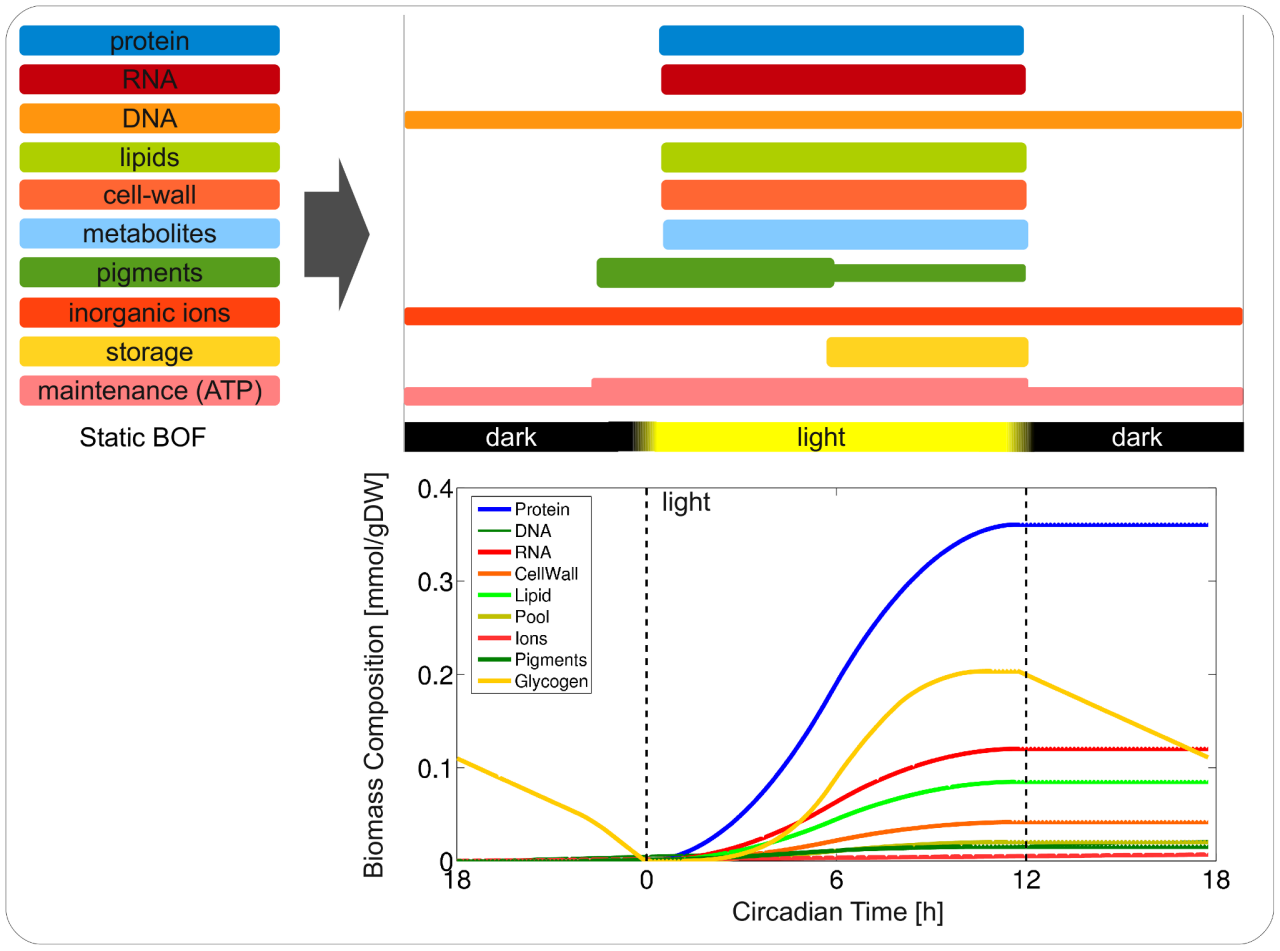
A time-dependent biomass objective function. The upper panel shows time-dependent objectives as inferred from literature and transcript data over a full diurnal cycle. The lower panel shows a simulation of the newly synthesized biomass components as a function of circadian time (CT). CT = 0 h marks sunrise.

Based on these empirical observations, we constructed a putative time-resolved biomass objective function, shown in Figure 6, to simulate diurnal metabolic activity of the metabolism of *Synechocystis* sp. PCC 6803. Instead of a single BOF, the biomass components are synthesized according to the following rules: We assume constant rate of uptake of micronutrients (inorganic ions). Likewise, DNA is assumed to be synthesized at a constant rate. All other biomass components are represented by a factor in a BOF that is optimized according to light availability. To mimic results on pigment fluorescence, the factor for pigments increases two hours before sunrise and decreases again after noon. In contrast, the factor corresponding to storage synthesis only increases after noon. In addition, ATP requirements for protein synthesis are included in the BOF, corresponding to an increased demand of ATP during growth. Similar to the dark simulation discussed above, the solution assumes a residual respiration also during periods of light availability, implemented as a lower bound for the corresponding flux. Formation of superoxide and the Mehler reaction is light dependent. During night glycogen is used to drive cellular respiration and maintenance. Biomass accumulation makes use of dynamic FBA [63], computational details are provided in the Materials and Methods.

**Figure 7:**
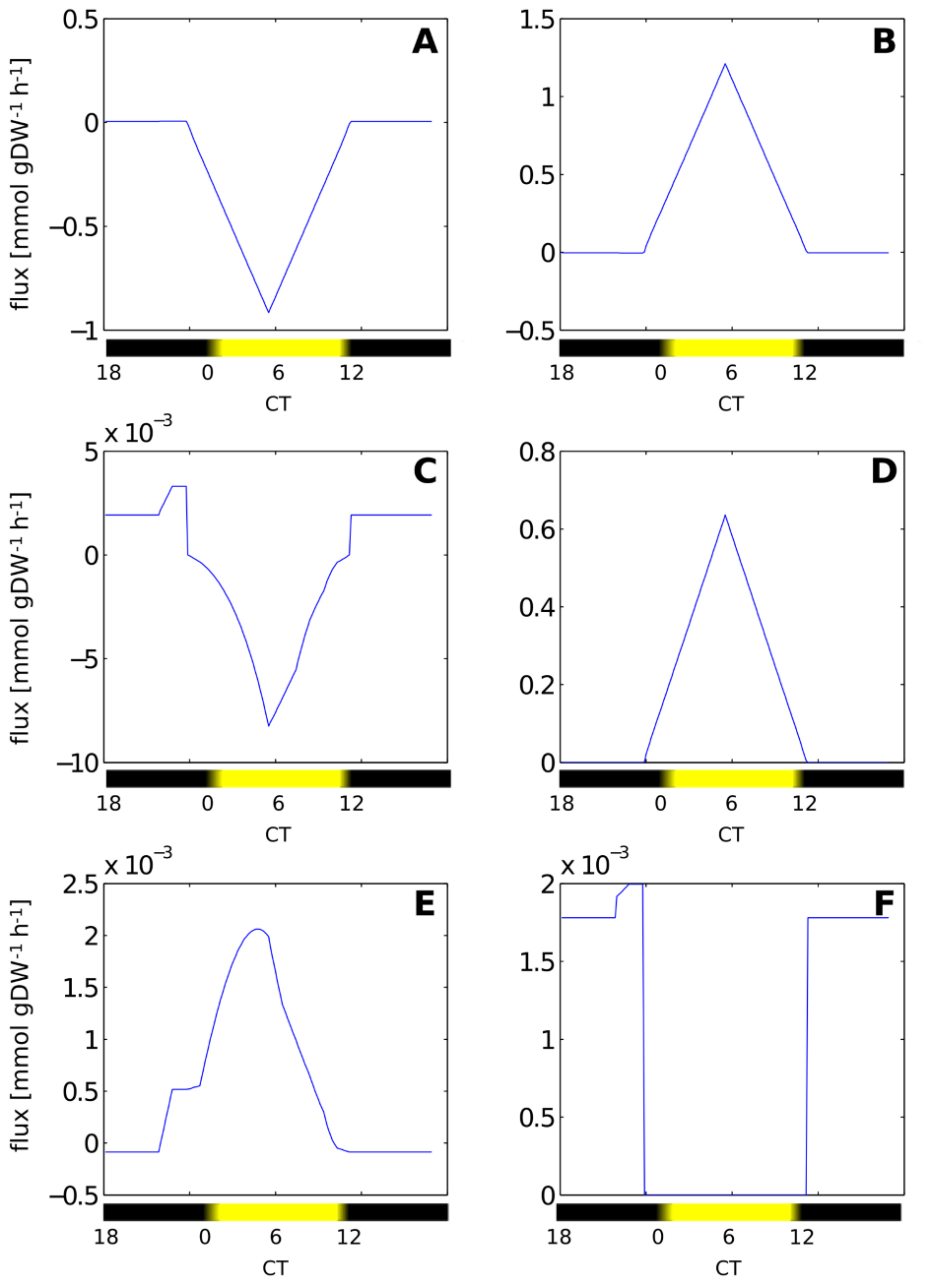
Selected metabolic fluxes over a full diurnal cycle. Shown is (A) oxygen uptake, (B) phosphoglycerate kinase, (C) phosphoglucomutase, (D) carboxylation of RuBisCO, (E) CDP kinase and (F) succinic semialdehyde dehydrogenase (TCA bypass).

The results of a full diurnal simulations are shown in Figure 6. We observe complex transitions in metabolic flux over the full 24 h period, shifting from respiration-dominated night metabolism, to biosynthesis and growth during the day. By construction, pigments rise early in the morning and remain approximately constant after noon. Glycogen content sharply increases during the second half of the day and is utilized during night. The time-courses of selected metabolic fluxes are provided in Figure 7. Shown in Figure 7A is the net-uptake of oxygen that is positive in the absence of light and follows light availability during the day; Figure 7B shows the flux through the RuBisCO reaction that describes photosynthetic activity and matches availability of energy. Figure 7C shows flux through the phosphoglycerate kinase with small negative flux during night corresponding to the utilization of glycogen and large positive flux during the day corresponding to the regeneration of the Calvin-Benson cycle. Figure 7D shows the interconversion of G1P and G6P with a positive flux corresponding to glycogen degradation and negative flux during the day, corresponding to glycogen, as well as lipid, synthesis. Figure 7E shows the interconversion of CTP and CDP corresponding to synthesis of pigments and DNA. Finally, Figure 7F shows the succinate-semialdehyde dehydrogenase that closes the TCA cycle and exhibits positive flux during night and no flux otherwise. A depiction of diurnal temporal changes over the entire network is provided as Figure S3.

### Conclusions

Phototrophic microorganisms hold great promises as a resource to generate high-value products and biofuels using only atmospheric carbon dioxide, sunlight, and some minerals. In this respect, cyanobacteria have attracted recent attention as a possible chassis for the generation of third generation biofuels. We presented an updated and extended genome-scale reconstruction for the unicellular cyanobacterium *Synechocystis* sp. PCC 6803. The updated reconstruction of the metabolic network is based on several existing reconstructions and incorporates novel results with respect to the cyanobacterial TCA cycle, an alleged glyoxylate shunt, as well as the role of photorespiration in cellular growth. The model includes various aspects specific for phototrophic metabolism, such as a light-dependent generation of reactive oxygen species. In addition to the model itself, which is encoded and made available in SBML format, we prepared a detailed graphical overview to facilitate discussion also among non-experts. The focus of our analysis was phototrophic growth of the organism, in particular the functional consequences of dissenting or unclear pathway topologies.

Indeed, despite several recent reconstructions, and the extensive biochemical literature available for *Synechocystis* sp. PCC 6803, several key reactions steps remain unclear. We evaluated several alternative possibilities to close the cyanobacterial TCA cycle, and compared biomass yield for different scenarios. An evolutionary advantage of the GABA shunt over the traditional OGDH, as proposed by Nogales et *al.* [17], could not be confirmed. However, the recently identified TCA bypass of Zhang and Bryant [49], as well as the GABA-shunt, require considerably smaller investment in enzyme synthesis than the OGDH complex – and therefore might be evolutionary advantageous for unicellular organisms that primarily rely on phototrophic growth. To reconcile existing reconstructions, we experimentally tested for the presence of an alleged glyoxylate shunt, included within several recent reconstruction based on biochemical evidence. We could not confirm enzymatic activity of the isocitrate lyase under the conditions tested. Neither was *Synechocystis* sp. PCC 6803 able to grow on acetate in the presence of DCMU to inhibit PSII and water oxidation and thus linear electron transport, but not PSI cyclic electron transport and ATP generation. Both facts strongly suggest the absence of a functional glyoxylate shunt.

As a feature that is specific to phototrophic organisms, the re-organization of metabolism in alternating diurnal light/dark cycles was demonstrated. As yet, almost all existing reconstructions have focussed on an evaluation of hetero-, mixo-, or phototrophic growth under constant light, with little reference to the actual environmental conditions the organism experiences. While such a simulation is clearly in its infancy, data on gene expression and physiological properties allow to describe a basic diurnal metabolic cycle of the organism. We are confident that similar computational approaches are required to obtain a better understanding of principles and trade-offs during phototrophic growth. In addition to the evaluation of a diurnal cycle, our reconstruction highlights several open questions with respect to cyanobacterial metabolism that deserve future attention. In particular, dark metabolism, as well as the interplay between oxygenic photosynthesis and aerobic respiration taking place in a single compartment are still insufficiently understood. We also conjecture that large-scale metabolic network modelling has to move beyond the stoichiometric reconstruction process itself and increasingly has to take into account additional biophysical constraints, such as photorespiration and the generation of reactive oxygen species, as well as suboptimal flux distributions to elucidate and explain observed metabolic behavior.

## Materials and Methods

### Network reconstruction and gaps

Although several reconstructions of the cyanobacterium *Synechocystis* sp. PCC 6803 have recently become available, only few attempts have been made to systematize the missing metabolic knowledge. Indeed, several aspects of the metabolic network and its main synthesis pathways are still insufficiently understood. For example, within the current reconstruction, the amino acids methionine and asparagine lack a complete synthesis pathway. To ensure viability in silico, the synthesis steps from *Microcystis aeruginosa* have been adopted for the synthesis of methionine. Asparagine is assumed to be synthesized from aspartate via an asparagine synthetase (EC 6.3.5.4). Synthesis pathways for all remaining amino acids are annotated, the putative enzymatic steps for serine and glycine are discussed in more detail below. Cyanobacteria utilize glycogen, cyanophycin and polyhydroxybutyrate (PHB) as storage compounds. However, the enzymatic steps necessary for breakdown of internal PHB are not known, even though the compound is detected [64]. Enzymatic steps for the synthesis of several components of the cell wall are not annotated, such as UDP-glucose and glycerolipids. Likewise, the annotation of the synthesis pathways of vitamin B6 and B12 are fragmentary. More fundamental, it is not fully known whether plastoquinone or ubiquinone is used within the electron transport chain (ETC). While the synthesis pathway of plastoquinone is partially present in *Synechocystis* sp. PCC 6803, a knock-out showed that its absence had no effect on photosynthetic function [65]. Therefore, there might be an additional pathway for plastoquinone or the organism may use ubiquinone. An alternative pathway for plastoquinone was recently suggested [66]. Further missing enzymatic steps were identified using BLAST search in available repositories, starting with related cyanobacterial strains [67]. In addition, primary biochemical literature was screened to identify possible alternative enzymatic routes. A list of missing or unclear enzymatic steps and necessary additions to ensure viability of the organism in silico is included within Supplementary Table S1.

Prior to conversion into final simulation files, the network was tested for elemental and charge balances using the COBRA toolbox [30] and the toolbox SubLiminal [68]. In case of unclear specificity of a reaction for NAD/NADH or NADP/NADPH, only the latter was included. Within the section ‘Metabolic flux during periods of darkness’, the glutamate dehydrogenase (GDH) reaction was assumed to be irreversible to avoid metabolic cycles that compensate for the lack of the transhydrogenase. The reconstructed network file (Supplemental Dataset S1) is compliant with MIRIAM. When available, all metabolites are referenced by their corresponding CheEBI ID [69]. We note that the intracellular pH of *Synechocystis* sp. PCC 6803 changes under diurnal conditions, from approx 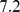 to 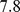 [70]. For simplicity, we use the values 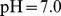 as a reference condition. The network file used for simulation with the COBRA toolbox is included as Supplemental Dataset S2.

### Overview on existing reconstructions

While metabolic reconstruction of phototrophic organisms are still underrepresented as compared to heterotrophic microorganisms, recently a number of cyanobacterial reconstructions have become available. The first application of FBA on cyanobacterial metabolism was performed by Shastri and Morgan [10], followed by an extension of the model by Hong and Lee [11]. Both reconstructions are comparatively small, with a focus on central metabolism. The models contain an incomplete TCA cycle and an alleged glyoxylate shunt. The first large-scale model was provided by Fu [12]. However, the reconstruction only involved little manual curation and actual flux was restricted to central metabolism, mainly because of the use of a restricted biomass objective function. The model also included an alleged glyoxylate shunt. An improved reconstruction was presented by Knoop et al. [13], albeit still limited in size. The reconstruction went beyond pathway repositories and included the first detailed representation of the photorespiratory pathways in a metabolic reconstruction, did not include the glyoxylate shunt. Instead the GABA shunt was active to close the incomplete TCA cycle. Shortly afterwards, three additional reconstructions were published [14]–[16], each again incorporating an incomplete TCA cycle and an alleged glyoxylate shunt. The analysis of Yoshikawa et al. [16] also compares the solutions of Knoop et al. [13] and Montagud et al. [14] under conditions of heterotrophic growth. A further reconstruction was presented by Nogales et al. [17], making use of an improved biomass function derived from literature data. The reconstruction does not include a glyoxylate shunt but does also not incorporate the TCA bypass of Zhang and Bryant [49]. Subsequently, a reconstruction of *Cyanothece* sp. ATCC 51142 [71], as well as of *Synechococcus* sp. PCC 7002 [72] was presented. Recently, a comparison of the metabolic potential of the strains *Cyanothece* sp. ATCC 51142 and *Synechocystis* sp. PCC 6803 was performed [18]. Network reconstruction is also increasing performed algorithmically [73], based on genomic similarity [67]. However, in such cases, manual reconstructions remain the gold standard to test the validity of automatically generated networks. A tabular overview of existing reconstructions is provided as Supplemental Text S2.

### Optimality of the TCA bypass

Nogales et al. [17] argue that having a GABA shunt instead of a complete TCA cycle may represent an evolutionary advantage in autotrophic conditions at the expense of reduced growth performance in heterotrophic conditions. This hypothesis was tested by alternatively forcing flux through a potential OGDH complex, as well through the glutamate synthase in autotrophic and heterotrophic conditions. Forced flux through the OGDH complex leads to an immediate and strong reduction of growth under autotrophic conditions, whereas forced flux through the glutamate synthase only leads to a slight reduction of autotrophic growth at high flux rates. However, as detailed in the main text, since autotrophic growth does not require a closed TCA cycle, forced flux through the OGDH complex therefore represents an unnecessary metabolic burden under this condition. On the other hand, the glutamate synthase carries non-zero flux also during autotrophic growth. To introduce a forced lower bound is therefore expected to have no immediate impact and only affects growth at if a high flux is forced. Indeed, if flux is forced instead through the succinic semialdehyde dehydrogenase, rather than the glutamate synthase, similar results as for the OGDH complex are obtained. We suggest that a possible explanation for the absence of the OGDH complex, despite the higher stoichiometry yield of the respective pathway, are the high synthesis requirements for the OGDH multiprotein complex.

### Time-resolved flux balance analysis

For the modelling of a full diurnal cycle under usage of the flux balance analysis we assume a doubling time of about 24 h. Since FBA estimates the flux towards biomass components, given in mmol h^−1^ gDW^−1^, we integrated the flux values over 24 h to get an estimate of the total amount of each component during one diurnal cycle. Based on these given amounts, we postulated a scenario for a full diurnal cycle. We assume a continuously synthesis of the biomass components ‘DNA’ and ‘inorganic ions’. The components ‘Protein’, ‘RNA’, ‘Cell wall’ and ‘lipids’ are only synthesized during the light phase of the simulation. The synthesis of pigments starts two hours before the beginning of the light phase and ends two hours before the start of the dark phase. To account for the demand for energy during the dark phase, when no light is available, we assume that glycogen, synthesized and accumulated from the middle of the light phase on, is used as storage compound. During the light phase a steady respiration rate of the cytochrome c oxidase of 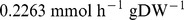 is assumed, which corresponds to about 10% of the maximally photosynthetically produced 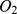, as well as a general ATP consumption for cellular maintenance of 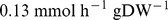. In the absence of light, the basal respiration rate is decreased to only 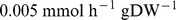 and ATP production is set as objective function. The full diurnal period was subdivided into 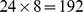 steps. For each step a new biomass function was assigned. The transition between different biomass configurations, as outlined above, was smoothened by dividing the difference of the weight factors within the BOF by the amount of steps between the configurations and changing them stepwise. The time-dependent BOF is provided in Supplemental Table S3. Light was assumed to follow a triangular shape, starting at circadian time 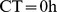 and peaking at 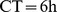 with a value of 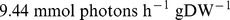.

### The glyoxylate shunt: experimental validation

#### Growth

*Synechocystis* PCC 6803 was cultivated in liquid BG11 medium buffered with HEPES/KOH (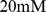, 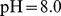) in glass bottles, gassed with air (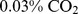) and continuously illuminated by cool white flourescent light of 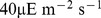.

#### Preparation of cell-free extract

Cells of 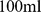 cell culture were harvested during the late logarithmic phase of growth, washed two times with buffer A (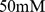 Tris/HCl, 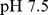, 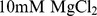, 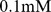 EDTA) and resuspended in 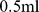 buffer A with 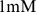 DTT. Cells were broken with glass beads in a Retsch cell mill at 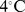 for 10 min. Glass beads were removed by centrifugation for 5 min and 2000 rcf. Cell wall debris was removed by centrifugation at 18000 rcf for 20 min and 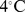, yielding the crude cell extract. For the removal of cellular metabolites these extract were passed over a PD-10 column (GE Healthcare) with buffer A (including 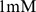 DTT) as elution buffer, yielding filtered cell extract.

#### Isocitrate lyase assay

Isocitrate lyase was assayed by the standard procedure [47]. Measurement have been carried out at room temperature in 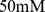 MOPS, pH 7, 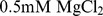, 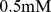 EDTA-Na2 (buffer B) including cell extract corresponding to 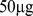 protein per ml reaction volume. The reaction was started by the consecutive addition of phenylhydrazin (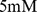), isocitrate (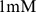) and NADP (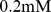) and changes in absorbance at 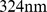 are measured over 30 minutes. Formation of the glyoxylic acid phenylhydrazone is calculated using a molar extinction coefficient at 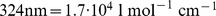. Protein was determinated by the Coomassie blue binding assay.

#### Determination of 2-Oxoglutarate

Buffer B, with the same protein concentration as used in the isocitrate lyase assay, was incubated with isocitrate (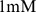) for 12 hours at ambient temperature. Then protein was denatured by means of heat treatment and removed by centrifugation. The clear supernatant was used for determination of 2-oxoglutarate by the UV-spectrophotometric method which uses NADH and glutamate dehydrogenase [74].

#### Spot assays

Cells of the logarithmic growth phase with a chlorophyll content of 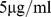 were plated in different dilutions (1∶1 undiluted, 1∶10, 1∶100) on BG11 agar, pH 8.0 containing sodium acetate in a final concentration of 0.2% w/v and photosynthesis inhibitor DCMU (3-(3,4-dichlorophenyl)-1,1-dimethylurea) in a final concentration of 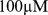. Plates were incubated under continuous illumination with white light of 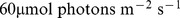 at 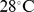 for 6 days. Respective controls were conducted without the addition of DCMU and sodium acetate, respectively.

### Analysis of transcripts

*Synechocystis* sp. PCC 6803 was grown in BG11-medium at 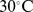 under continuous illumination with white light of 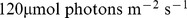 and a continuous stream of air. Cultures were synchronized with three cycles of light/dark 12 h∶12 h prior sampling. Over a 24 h time course, 6 samples for RNA isolation were taken 30 minutes before and after light is switched off, (sample 1,2), 30 minutes before midnight (sample 3), 30 minutes before and after light onset (sample 4,5) and 30 minutes before noon (sample 6). Two replicates were prepared from two synchronously growing cultures. The microarray design and hybridization procedure have been described previously [75]. The “Agilent Feature Extraction Software 10.5.1.1” was used for extraction of the spot intensities. In accordance to findings in other cyanobacterial species [56], [76], [77] we observe a large number of genes with diurnal expression patterns and a global trend towards higher gene expression levels over the subjective day. We adopted an approach similar to Calza et al. [78] by first finding a set of observed expression profiles which exhibit the lowest degree of diurnal oscillation. We assume, that the expression of this set of genes (Least-Oscillating-Set or LOS) remains unchanged and variation exclusively reflects technical variation. The Loess curve calculated between LOS gene expressions in each microarray and the LOS gene mean expressions is then applied to the entire microarray. The oscillation strength of an expression profile was measured by the power spectral density corresponding to a frequency of 1/d (pd). By repeated shuffling and pd computation for 105 times, we obtained a p-value 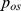 for randomly observing each genes diurnal oscillatory behavior. Genes with 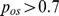 where included in the LOS, yielding a total of 1173 genes. The phase for each expression profile was also calculated using the Fourier transformation. Details on experimental and computational analysis are described in Lehmann et al. [79].

### Abbreviations used

The following abbreviations are used in Figure 2: 2OG (2-Oxogluterate), 2Oiv (2-Oxoisovalerate), 2PGL (2-Phosphoglycolate), 3PG (3-Phospho-glyceroyl phosphate), 4AB (4-Aminobutanoate), 5PrPP (5-Phospho-ribose 1-diphosphate), A4Sa (Aspartate 4-semialdehyde), AcCoA (Acetyl-CoA), Ace (Acetate), AceP (Acetyl phosphate), ADP (Adenosine 5′-diphosphate), AIR (Aminoimidazole ribotide), Ala (Alanine), Arg (Arginine), Asn (Asparagine), Asp (Aspartate), ATP (Adenosine 5′-triphosphate), ATPase (ATP synthase), bCaro (beta-Carotene), Chlp (Chlorophyll a), Chor (Chorismate), Cit (Citrate), COX (Cytochrome c oxidase), Cys (Cysteine), Cyt b6f (Cytochrome b6-f complex), DGDG (Digalactosyl-diacylglycerol), DHAP (Dihydroxyacetone phosphate), DX5P (1-Deoxy-xylulose 5-phosphate), E4P (Erythrose 4-phosphate), Echi (Echinenone), F6P (Fructose 6-phosphate), FAD (Flavin adenine dinucleotide), FBP (Fructose 1,6-bisphosphate), FNR (Ferredoxin-NADP reductase), Fum (Fumarate), G1P (Glucose 1-phosphate), G6P (Glucose 6-phosphate), GAP (Glyceraldehyde 3-phosphate), gCaro (gamma-Carotene), GgPP (Geranylgeranyl diphosphate), GL (Glycolate), Gln (Glutamine), Glu (Glutamate), GLX (Glyxoylate), Gly (Glycine), GSH (Glutathione), His (Histidine), Hser (Homoserine), Icit (Isocitrate), Ile (Isoleucine), IpPP (Isopentenyl diphosphate), Leu (Leucine), LpAD (Lipid A disaccharide), Lys (Lysine), MaCoA (Malonyl-CoA), Mal (Malate), mDom (meso-2,6-Diaminopimelate), Met (Methionine), MGDG (Monogalactosyl-diacylglycerol), NAD (Nicotinamide adenine dinucleotide), NADP (Nicotinamide adenine dinucleotide phosphate), NDH (NADPH dehydrogenase), OA (Oxaloactete), PC (Plastocyanin PEP (Phosphoenolpyruvate), PepGlc (Peptidoglycan), PG (Phosphatidylglycerol), PG2 (Glycerate 2-phosphate), PG3 (Glycerate 3-phosphate), Phe (Phenylalanine), Pho (Phosphate), Phyq (Phylloquinone), Ppg (Protoporphyrinogen), PPP (Phytyl diphosphate), PQ (Plastoquinone), Prep (Prephenate), Pro (Proline), PS I (Photosystem I), PS II (Photosystem II), Ptd (Phosphatidate), Ptsn (Putrescine), Pyr (Pyruvate), R5P (Ribose 5-phosphate), Rbfv (Riboflavin), Ru5P (Ribulose 5-phosphate), RuBP (Ribulose 1,5-bisphosphate), S7P (Sedoheptulose 7-phosphate), SAM (S-Adenosylmethioninamine), SBP (Sedoheptulose 1,7-bisphosphate), ScCoA (Succinyl-CoA), SDH (Succinate dehydrogenase), Ser (Serine), Spmd (Spermidine), SPP (Solanyl diphosphate), SQDG (Sulfoquinovosyldiacylglycerol), Ssa (Succinate semialdehyde), Suc (Succinate), Sul (Sulfur), THF (Tetrahydrofolate), ThPP (Thiamin diphosphate), Thr (Threonine), Toco (Tocopherol), Trp (Tryptophan), Tyr (Tyrosine), Upg III (Uroporphyrinogen III), UpPP (Undecaprenyl diphosphate), Val (Valine), Vit B12 (Vitamin B12), Vit B6 (Vitamin B6), X5P (Xylulose 5-phosphate), Zea (Zeaxanthin).

## Supporting Information

Dataset S1 An annotated SBML file encoding the metabolic network of *Synechocystis* sp. PCC 6803.
(XML)

Dataset S2 The reconstructed network suitable for simulation with the COBRA toolbox.
(XML)

Figure S1 A detailed graphical overview of the metabolic network. Best printed in A0 format.
(PDF)

Figure S2 Phase-sorted expression profiles of metabolic genes in *Synechocystis* sp. PCC 6803 as a function of circadian time (CT). Two independent replicates are shown consecutively. The majority of transcripts peaks during day. A list of phase-sorted transcripts is provided as Supplemental Table S4.
(PNG)

Figure S3 Clustergram of metabolic fluxes over a full diurnal cycle. Shown are reactions that carry a flux along the diurnal cycle. Isoreactions are combined into a single reaction.
(EPS)

Figure S4 Positive control of ICL activity. Isocitrate lyase activity in cell free extracts of *E. coli* was measured in phenylhydrazin reaction buffer. Increase in A324 nm after adding of the substrate isocitrate (IC) with an end concentration of 1 mM shows the formation of glyoxylate phenylhydrazon.
(TIFF)

Table S1 An excel sheet containing the metabolic network of *Synechocystis* sp. PCC 6803.
(XLS)

Table S2 A list of annotated enzymes that are not part of the core network.
(XLS)

Table S3 Results of Flux Balance Analysis, including simulated flux values for light and dark metabolism, flux variability and diurnal variation.
(XLS)

Table S4 A list of phase-sorted transcripts.
(XLS)

Table S5 Amino acid requirements for the TCA cycle and its bypass.
(XLS)

Text S1 Positive control of ICL activity.
(PDF)

Text S2 Summary of existing reconstructions.
(PDF)

## References

[1] Knoll AH (2008) Cyanobacteria and Earth History in The Cyanobacteria: Molecular Biology, Genomics and Evolution. Herrero A, Flores E, editors. Caister Academic Press. pp. 1–19.

[2] Ducat DC, Way JC, Silver PA (2011) Engineering cyanobacteria to generate high-value products. Trends Biotechnol 29: 95–103. 21211860 10.1016/j.tibtech.2010.12.003

[3] Hess WR (2011) Cyanobacterial genomics for ecology and biotechnology. Curr Opin Microbiol 14: 608–14. 21840247 10.1016/j.mib.2011.07.024

[4] Atsumi S, Higashide W, Liao JC (2009) Direct photosynthetic recycling of carbon dioxide to isobutyraldehyde. Nat Biotechnol 27: 1177–1180. 19915552 10.1038/nbt.1586

[5] Liu X, Sheng J, Curtiss R (2011) Fatty acid production in genetically modified cyanobacteria. Proc Natl Acad Sci U S A 108: 6899–6904. 21482809 10.1073/pnas.1103014108 PMC3084101

[6] Lindberg P, Park S, Melis A (2010) Engineering a platform for photosynthetic isoprene production in cyanobacteria, using Synechocystis as the model organism. Metab Eng 12: 70–79. 19833224 10.1016/j.ymben.2009.10.001

[7] Quintana N, der Kooy FV, de Rhee MDV, Voshol GP, Verpoorte R (2011) Renewable energy from cyanobacteria: energy production optimization by metabolic pathway engineering. Appl Microbiol Biotechnol 91: 471–490. 21691792 10.1007/s00253-011-3394-0 PMC3136707

[8] Lan EI, Liao JC (2012) ATP drives direct photosynthetic production of 1-butanol in cyanobacteria. Proc Natl Acad Sci U S A 109: 6018–6023. 22474341 10.1073/pnas.1200074109 PMC3341080

[9] Rosgaard L, de Porcellinis AJ, Jacobsen JH, Frigaard NU, Sakuragi Y (2012) Bioengineering of carbon fixation, biofuels, and biochemicals in cyanobacteria and plants. J Biotechnol 162 1: 134–47. 22677697 10.1016/j.jbiotec.2012.05.006

[10] Shastri AA, Morgan JA (2005) Flux balance analysis of photoautotrophic metabolism. Biotechnol Prog 21: 1617–1626. 16321043 10.1021/bp050246d

[11] Hong SJ, Lee CG (2007) Evaluation of central metabolism based on a genomic database of Synechocystis PCC 6803. Biotechnology and Bioprocess Engineering 12: 165–173.

[12] Fu P (2009) Genome-scale modeling of Synechocystis sp. PCC 6803 and prediction of pathway insertion. J Chem Technol Biotechnol 84: 473483.

[13] Knoop H, Zilliges Y, Lockau W, Steuer R (2010) The metabolic network of Synechocystis sp. PCC 6803: systemic properties of autotrophic growth. Plant Physiol 154: 410–422. 20616194 10.1104/pp.110.157198 PMC2938163

[14] Montagud A, Navarro E, de Cordoba PF, Urchueguia JF, Patil KR (2010) Reconstruction and analysis of genome-scale metabolic model of a photosynthetic bacterium. BMC Syst Biol 4: 156. 21083885 10.1186/1752-0509-4-156 PMC3009638

[15] Montagud A, Zelezniak A, Navarro E, de Cordoba PF, Urchueguia JF, et al (2011) Flux coupling and transcriptional regulation within the metabolic network of the photosynthetic bacterium Synechocystis sp. PCC 6803. Biotechnol J 6: 330–342. 21226012 10.1002/biot.201000109

[16] Yoshikawa K, Kojima Y, Nakajima T, Furusawa C, Hirasawa T, et al (2011) Reconstruction and verification of a genome-scale metabolic model for Synechocystis sp. PCC 6803. Appl Microbiol Biotechnol 92: 347–358. 21881889 10.1007/s00253-011-3559-x

[17] Nogales J, Gudmundsson S, Knight EM, Palsson BO, Thiele I (2012) Detailing the optimality of photosynthesis in cyanobacteria through systems biology analysis. Proc Natl Acad Sci U S A 109: 2678–2683. 22308420 10.1073/pnas.1117907109 PMC3289291

[18] Saha R, Verseput AT, Berla BM, Mueller TJ, Pakrasi HB, et al (2012) Reconstruction and comparison of the metabolic potential of cyanobacteria Cyanothece sp. ATCC 51142 and Synechocystis sp. PCC 6803. PLoS One 7: e48285. 23133581 10.1371/journal.pone.0048285 PMC3487460

[19] Thiele I, Palsson BO (2010) A protocol for generating a high-quality genome-scale metabolic reconstruction. Nat Protoc 5: 93–121. 20057383 10.1038/nprot.2009.203 PMC3125167

[20] Hucka M, Finney A, Sauro HM, Bolouri H, Doyle JC, et al (2003) The systems biology markup language (SBML): a medium for representation and exchange of biochemical network models. Bioinformatics 19: 524–531. 12611808 10.1093/bioinformatics/btg015

[21] Orth JD, Thiele I, Palsson BO (2010) What is flux balance analysis? Nat Biotechnol 28: 245–248. 20212490 10.1038/nbt.1614 PMC3108565

[22] Steuer R, Knoop H, Machne R (2012) Modelling cyanobacteria: from metabolism to integrative models of phototrophic growth. Journal of Experimental Botany 63: 2259–74. 22450165 10.1093/jxb/ers018

[23] Cooley JW, Vermaas WF (2001) Succinate dehydrogenase and other respiratory pathways in thylakoid membranes of Synechocystis sp. strain PCC 6803: capacity comparisons and physiological function. J Bacteriol 183: 4251–4258. 11418566 10.1128/JB.183.14.4251-4258.2001 PMC95315

[24] Vermaas W (2001) Photosynthesis and respiration in cyanobacteria. Encyclopedia of Life Sciences 1–7.

[25] Yao DCI, Brune DC, Vermaas WFJ (2012) Lifetimes of photosystem I and II proteins in the cyanobacterium Synechocystis sp. PCC 6803. FEBS Lett 586: 169–173. 22197103 10.1016/j.febslet.2011.12.010

[26] Helman Y, Tchernov D, Reinhold L, Shibata M, Ogawa T, et al (2003) Genes encoding A-type avoproteins are essential for photoreduction of O2 in cyanobacteria. Curr Biol 13: 230–235. 12573219 10.1016/s0960-9822(03)00046-0

[27] Allahverdiyeva Y, Ermakova M, Eisenhut M, Zhang P, Richaud P, et al (2011) Interplay between avodiiron proteins and photorespiration in Synechocystis sp. PCC 6803. J Biol Chem 286: 24007–24014. 21602273 10.1074/jbc.M111.223289 PMC3129182

[28] Tchernov D, Silverman J, Luz B, Reinhold L, Kaplan A (2003) Massive light-dependent cycling of inorganic carbon between oxygenic photosynthetic microorganisms and their surroundings. Photosynth Res 77: 95–103. 16228368 10.1023/A:1025869600935

[29] Helman Y, Barkan E, Eisenstadt D, Luz B, Kaplan A (2005) Fractionation of the three stable oxygen isotopes by oxygen-producing and oxygen-consuming reactions in photosynthetic organisms. Plant Physiol 138: 2292–2298. 16040650 10.1104/pp.105.063768 PMC1183415

[30] Schellenberger J, Que R, Fleming RMT, Thiele I, Orth JD, et al (2011) Quantitative prediction of cellular metabolism with constraint-based models: the COBRA toolbox v2.0. Nat Protoc 6: 1290–1307. 21886097 10.1038/nprot.2011.308 PMC3319681

[31] Hoppe A, Hoffmann S, Gerasch A, Gille C, Holzhuetter HG (2011) FASIMU: flexible software for flux-balance computation series in large metabolic networks. BMC Bioinformatics 12: 28. 21255455 10.1186/1471-2105-12-28 PMC3038154

[32] Young JD, Shastri AA, Stephanopoulos G, Morgan JA (2011) Mapping photoautotrophic metabolism with isotopically nonstationary (13)C flux analysis. Metab Eng 13: 656–65. 21907300 10.1016/j.ymben.2011.08.002 PMC3210925

[33] Sonderegger M, Schumperli M, Sauer U (2004) Metabolic engineering of a phosphoketolase pathway for pentose catabolism in Saccharomyces cerevisiae. Appl Environ Microbiol 70: 2892–2897. 15128548 10.1128/AEM.70.5.2892-2897.2004 PMC404438

[34] Chinen A, Kozlov YI, Hara Y, Izui H, Yasueda H (2007) Innovative metabolic pathway design for efficient l-glutamate production by suppressing CO2 emission. J Biosci Bioeng 103: 262–269. 17434430 10.1263/jbb.103.262

[35] Yang C, Hua Q, Shimizu K (2002) Metabolic flux analysis in Synechocystis using isotope distribution from 13C-labeled glucose. Metab Eng 4: 202–216. 12616690 10.1006/mben.2002.0226

[36] Fischer E, Sauer U (2005) Large-scale in vivo flux analysis shows rigidity and suboptimal performance of Bacillus subtilis metabolism. Nat Genet 37: 636–640. 15880104 10.1038/ng1555

[37] Schuetz R, Zamboni N, Zampieri M, Heinemann M, Sauer U (2012) Multidimensional optimality of microbial metabolism. Science 336: 601–604. 22556256 10.1126/science.1216882

[38] Gründel M, Scheunemann R, Lockau W, Zilliges Y (2012) Impaired glycogen synthesis causes metabolic overow reactions and affects stress responses in the cyanobacterium Synechocystis sp. PCC 6803. Microbiology 158: 3032–3043. 23038809 10.1099/mic.0.062950-0

[39] Hackenberg C, Engelhardt A, Matthijs HCP, Wittink F, Bauwe H, et al (2009) Photorespiratory 2-phosphoglycolate metabolism and photoreduction of O2 cooperate in high-light acclimation of Synechocystis sp. strain PCC 6803. Planta 230: 625–637. 19578872 10.1007/s00425-009-0972-9 PMC2729987

[40] Bauwe H, Hagemann M, Kern R, Timm S (2012) Photorespiration has a dual origin and manifold links to central metabolism. Curr Opin Plant Biol 15: 269–275. 22284850 10.1016/j.pbi.2012.01.008

[41] Chiba Y, Oshima K, Arai H, Ishii M, Igarashi Y (2012) Discovery and analysis of cofactordependent phosphoglycerate mutase homologs as novel phosphoserine phosphatases in hydrogenobacter thermophilus. J Biol Chem 287: 11934–41. 22337887 10.1074/jbc.M111.330621 PMC3320941

[42] Huege J, Goetze J, Schwarz D, Bauwe H, Hagemann M, et al (2011) Modulation of the major paths of carbon in photorespiratory mutants of Synechocystis. PLoS One 6: e16278. 21283704 10.1371/journal.pone.0016278 PMC3025020

[43] Eisenhut M, Ruth W, Haimovich M, Bauwe H, Kaplan A, et al (2008) The photorespiratory glycolate metabolism is essential for cyanobacteria and might have been conveyed endosymbiontically to plants. Proc Natl Acad Sci U S A 105: 17199–17204. 18957552 10.1073/pnas.0807043105 PMC2579401

[44] Pearce J, Carr NG (1967) The metabolism of acetate by the blue-green algae, Anabaena variabilis and Anacystis nidulans. J Gen Microbiol 49: 301–313. 6077932 10.1099/00221287-49-2-301

[45] Eley JH (1988) Glyoxylate cycle enzyme activities in the cyanobacterium Anacystis nidulans. Journal of Phycology 24: 586588.

[46] Bandyopadhyay A, Elvitigala T, Welsh E, Stoeckel J, Liberton M, et al (2011) Novel metabolic attributes of the genus cyanothece, comprising a group of unicellular nitrogen-fixing Cyanothece. MBio 2: e00214–11. 21972240 10.1128/mBio.00214-11 PMC3187577

[47] Dixon G, Kornberg H (1959) Assay methods for key enzymes of the glyoxylate cycle. Biochem J 72: 2–3.

[48] Sweetlove LJ, Beard KFM, Nunes-Nesi A, Fernie AR, Ratcliffe RG (2010) Not just a circle: flux modes in the plant TCA cycle. Trends Plant Sci 15: 462–470. 20554469 10.1016/j.tplants.2010.05.006

[49] Zhang S, Bryant DA (2011) The tricarboxylic acid cycle in cyanobacteria. Science 334: 1551–1553. 22174252 10.1126/science.1210858

[50] Steinhauser D, Fernie AR, Arajo WL (2012) Unusual cyanobacterial TCA cycles: not broken just different. Trends Plant Sci 17: 503–9. 22658681 10.1016/j.tplants.2012.05.005

[51] Murphy GE, Jensen GJ (2005) Electron cryotomography of the E. coli pyruvate and 2-oxoglutarate dehydrogenase complexes. Structure 13: 1765–1773. 16338405 10.1016/j.str.2005.08.016

[52] Najdi TS, Hatfield GW, Mjolsness ED (2010) A ‘random steady-state’ model for the pyruvate dehydrogenase and alpha-ketoglutarate dehydrogenase enzyme complexes. Phys Biol 7: 16016. 20228444 10.1088/1478-3975/7/1/016016

[53] Schuster S, Pfeiffer T, Fell DA (2008) Is maximization of molar yield in metabolic networks favoured by evolution? J Theor Biol 252: 497–504. 18249414 10.1016/j.jtbi.2007.12.008

[54] Schuster S, de Figueiredo LF, Schroeter A, Kaleta C (2011) Combining metabolic pathway analysis with evolutionary game theory: explaining the occurrence of low-yield pathways by an analytic optimization approach. Biosystems 105: 147–153. 21620931 10.1016/j.biosystems.2011.05.007

[55] Johnson CH, Stewart PL, Egli M (2011) The cyanobacterial circadian system: from biophysics to bioevolution. Annu Rev Biophys 40: 143–167. 21332358 10.1146/annurev-biophys-042910-155317 PMC3093959

[56] Stoeckel J, Welsh EA, Liberton M, Kunnvakkam R, Aurora R, et al (2008) Global transcriptomic analysis of Cyanothece 51142 reveals robust diurnal oscillation of central metabolic processes. Proc Natl Acad Sci U S A 105: 6156–6161. 18427117 10.1073/pnas.0711068105 PMC2329701

[57] Cerven J, Nedbal L (2009) Metabolic rhythms of the cyanobacterium Cyanothece sp. ATCC 51142 correlate with modeled dynamics of circadian clock. J Biol Rhythms 24: 295–303. 19625731 10.1177/0748730409338367

[58] Stoeckel J, Jacobs JM, Elvitigala TR, Liberton M, Welsh EA, et al (2011) Diurnal rhythms result in significant changes in the cellular protein complement in the cyanobacterium Cyanothece 51142. PLoS One 6: e16680. 21364985 10.1371/journal.pone.0016680 PMC3043056

[59] McDermott JE, Oehmen CS, McCue LA, Hill E, Choi DM, et al (2011) A model of cyclic transcriptomic behavior in the cyanobacterium Cyanothece sp. ATCC 51142. Mol Biosyst 7: 2407–2418. 21698331 10.1039/c1mb05006k

[60] Waldbauer JR, Rodrigue S, Coleman ML, Chisholm SW (2012) Transcriptome and proteome dynamics of a light-dark synchronized bacterial cell cycle. PLoS One 7: e43432. 22952681 10.1371/journal.pone.0043432 PMC3430701

[61] Blazier AS, Papin JA (2012) Integration of expression data in genome-scale metabolic network reconstructions. Front Physiol 3: 299. 22934050 10.3389/fphys.2012.00299 PMC3429070

[62] Mori T, Binder B, Johnson CH (1996) Circadian gating of cell division in cyanobacteria growing with average doubling times of less than 24 hours. Proc Natl Acad Sci U S A 93: 10183–10188. 8816773 10.1073/pnas.93.19.10183 PMC38358

[63] Mahadevan R, Edwards JS, Doyle FJ (2002) Dynamic flux balance analysis of diauxic growth in Escherichia coli. Biophys J 83: 1331–1340. 12202358 10.1016/S0006-3495(02)73903-9 PMC1302231

[64] Taroncher-Oldenburg G, Nishina K, Stephanopoulos G (2000) Identification and analysis of the polyhydroxyalkanoate-specific beta-ketothiolase and acetoacetyl coenzyme A reductase genes in the cyanobacterium Synechocystis sp. strain PCC 6803. Appl Environ Microbiol 66: 4440–4448. 11010896 10.1128/aem.66.10.4440-4448.2000 PMC92322

[65] Daehnhardt D, Falk J, Appel J, van der Kooij TAW, Schulz-Friedrich R, et al (2002) The hydroxyphenylpyruvate dioxygenase from Synechocystis sp. PCC 6803 is not required for plastoquinone biosynthesis. FEBS Lett 523: 177–181. 12123828 10.1016/s0014-5793(02)02978-2

[66] Sadre R, Pfaff C, Buchkremer S (2012) Plastoquinone-9 biosynthesis in cyanobacteria differs from that in plants and involves a novel 4-hydroxybenzoate solanesyltransferase. Biochem J 442: 621–629. 22166075 10.1042/BJ20111796

[67] Beck C, Knoop H, Axmann IM, Steuer R (2012) The diversity of cyanobacterial metabolism: genome analysis of multiple phototrophic microorganisms. BMC Genomics 13: 56. 22300633 10.1186/1471-2164-13-56 PMC3369817

[68] Swainston N, Smallbone K, Mendes P, Kell D, Paton N (2011) The SuBliMinaL toolbox: automating steps in the reconstruction of metabolic networks. J Integr Bioinform 8: 186. 22095399 10.2390/biecoll-jib-2011-186

[69] Degtyarenko K, de Matos P, Ennis M, Hastings J, Zbinden M, et al (2008) ChEBI: a database and ontology for chemical entities of biological interest. Nucleic Acids Res 36: D344–D350. 17932057 10.1093/nar/gkm791 PMC2238832

[70] Falkner G, Horner F (1976) pH changes in the cytoplasm of the blue-green alga Anacystis nidulans caused by light-dependent proton flux into the thylakoid space. Plant Physiol 58: 717–718. 16659751 10.1104/pp.58.6.717 PMC542293

[71] Vu TT, Stolyar SM, Pinchuk GE, Hill EA, Kucek LA, et al (2012) Genome-scale modeling of light-driven reductant partitioning and carbon fluxes in diazotrophic unicellular cyanobacterium Cyanothece sp. ATCC 51142. PLoS Comput Biol 8: e1002460. 22529767 10.1371/journal.pcbi.1002460 PMC3329150

[72] Hamilton JJ, Reed JL (2012) Identification of functional differences in metabolic networks using comparative genomics and constraint-based models. Plos One 7 4: e34670. 22666308 10.1371/journal.pone.0034670 PMC3359066

[73] Vitkin E, Shlomi T (2012) MIRAGE: a functional genomics-based approach for metabolic network model reconstruction and its application to cyanobacteria networks. Genome Biol 13: R111. 23194418 10.1186/gb-2012-13-11-r111 PMC4053740

[74] Bergmeyer HU, editor(1985) Methods of Enzymatic Analysis, volume VII. VCH Verlagsgesellschaft.

[75] Georg J, Voss B, Scholz I, Mitschke J, Wilde A, et al (2009) Evidence for a major role of antisense RNAs in cyanobacterial gene regulation. Mol Syst Biol 5: 305. 19756044 10.1038/msb.2009.63 PMC2758717

[76] Kucho K, Okamoto K, Tsuchiya Y, Nomura S, Nango M, et al (2005) Global analysis of circadian expression in the cyanobacterium Synechocystis sp. strain PCC 6803. J Bacteriol 187: 2190–2199. 15743968 10.1128/JB.187.6.2190-2199.2005 PMC1064041

[77] Straub C, Quillardet P, Vergalli J, de Marsac NT, Humbert JF (2011) A day in the life of Microcystis aeruginosa strain PCC 7806 as revealed by a transcriptomic analysis. PLoS One 6: e16208. 21283831 10.1371/journal.pone.0016208 PMC3023806

[78] Calza S, Valentini D, Pawitan Y (2008) Normalization of oligonucleotide arrays based on the leastvariant set of genes. BMC Bioinformatics 9: 140. 18318917 10.1186/1471-2105-9-140 PMC2324100

[79] Lehmann R, Machne R, Georg J, Benary M, Axmann I, et al (2013) How cyanobacteria pose new problems to old methods: challenges in microarray time series analysis. BMC Bioinformatics 14: 133. 23601192 10.1186/1471-2105-14-133 PMC3679775

