# Supplementary figures and images for: Flux Balance Analysis of Cyanobacterial Metabolism: The Metabolic Network of Synechocystis sp. PCC 6803
Source: PLoS Comput Biol. 2013 Jun 27;9(6):e1003081. doi: 10.1371/journal.pcbi.1003081 (PMC3699288; doi:10.1371/journal.pcbi.1003081)

# The Metabolic Network of *Synechocystis* sp. PCC 6803

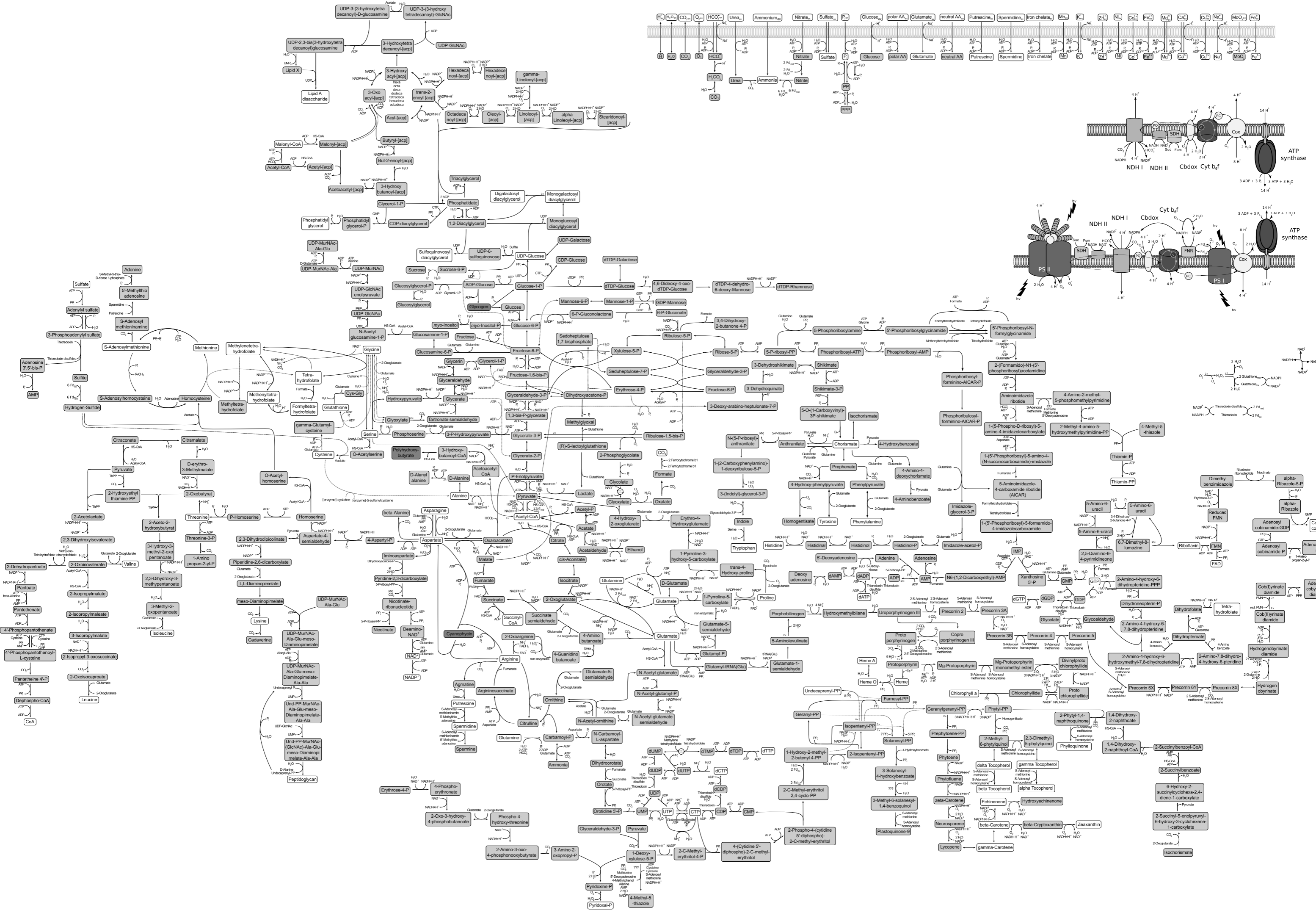

Supplement: Figure S1 — A detailed graphical overview of the metabolic network. Best printed in A0 format. (PDF) [file pcbi.1003081.s003.pdf]

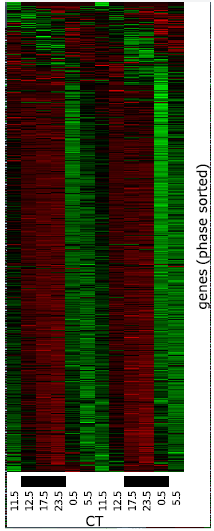

Supplement: Figure S2 — Phase-sorted expression profiles of metabolic genes in Synechocystis sp. PCC 6803 as a function of circadian time (CT). Two independent replicates are shown consecutively. The majority of transcripts peaks during day. A list of phase-sorted transcripts is provided as Supplemental Table S4. (PNG) [file pcbi.1003081.s004.png]

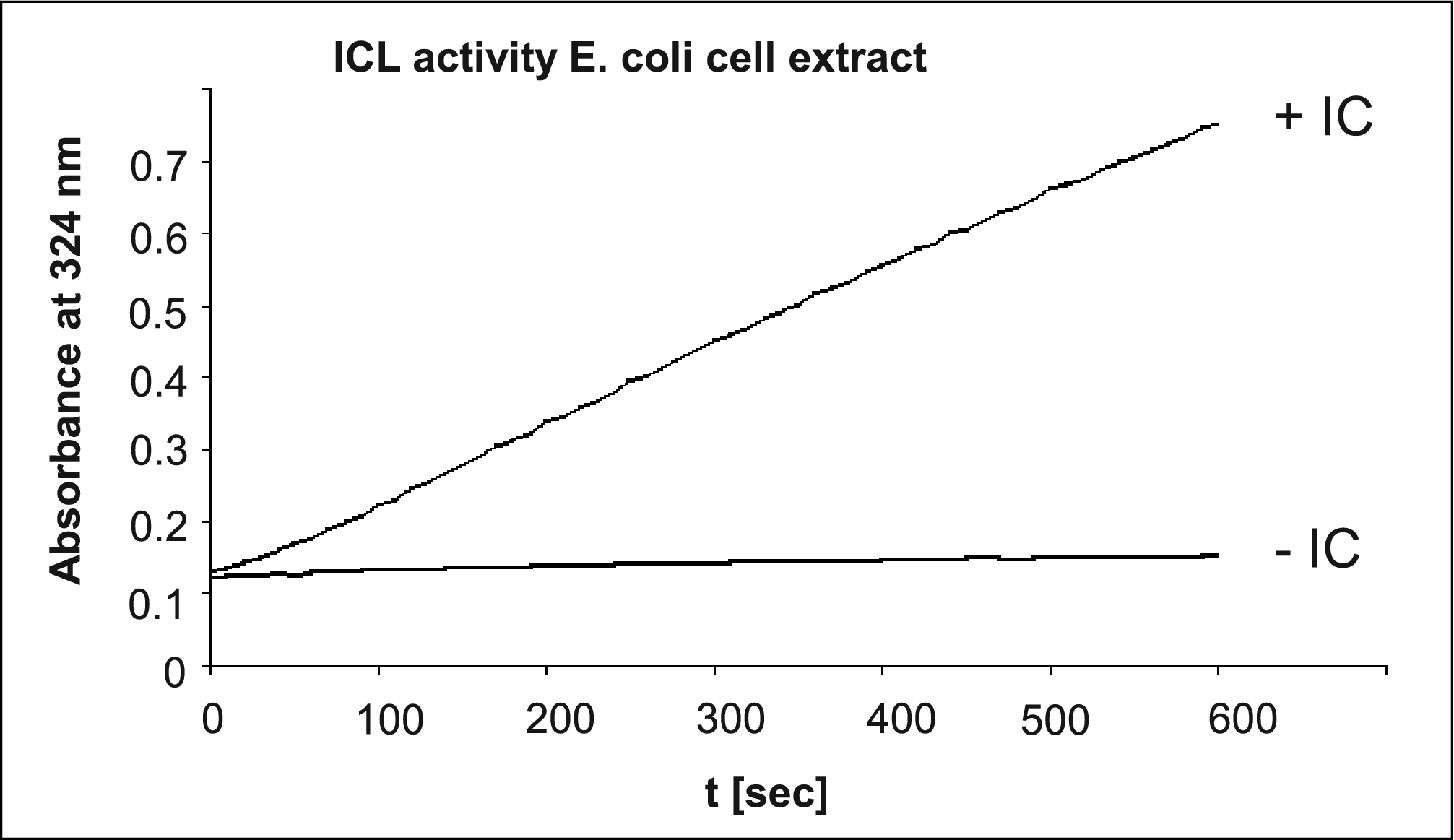

Supplement: Figure S4 — Positive control of ICL activity. Isocitrate lyase activity in cell free extracts of E. coli was measured in phenylhydrazin reaction buffer. Increase in A324 nm after adding of the substrate isocitrate (IC) with an end concentration of 1 mM shows the formation of glyoxylate phenylhydrazon. (TIFF) [file pcbi.1003081.s006.tiff]
