# Supplementary material for: Flux Balance Analysis of Cyanobacterial Metabolism: The Metabolic Network of Synechocystis sp. PCC 6803
Source: PLoS Comput Biol. 2013 Jun 27;9(6):e1003081. doi: 10.1371/journal.pcbi.1003081 (PMC3699288; doi:10.1371/journal.pcbi.1003081)
Supplement: Text S1 — Positive control of ICL activity. (PDF) [file pcbi.1003081.s012.pdf]

Supplemental Text S1:

Positive control for the isocitrate lyase (ICL) assay

As a positive control for the ICL assay we checked cell extracts from *Escherichia coli* K-12, grown on acetate as carbon source so as to induce the glyoxylate cycle enzymes (1). Under the experimental conditions used for the cyanobacterial extracts, ICL activities corresponding to ≈0.4 μmol glyoxylate formed min^{-1} mg^{-1} protein. See Figure S4 for results.

(1) S.R. Maloy & W.D. Nunn (1982) Genetic regulation of the glyoxylate shunt in *Escherichia coli* K-12. J. Bacteriol. **149**:173-180.

ICL activity under different culture conditions:

ICL-activity was not detectable in extracts from *Synechocystis* cells starved for a nitrogen source. This starvation results in an overflow of carbon in the cells comparable with mixotrophic growth conditions.

Measurements of ICL activity in cell extracts of *Synechococcus* PCC 7942, grown under a day/night regime and assayed in both growth phases, showed neither in the day nor in the night any detectable ICL activity. Corresponding data for *Synechocystis* are not available.
