# Supplementary material for: Flux Balance Analysis of Cyanobacterial Metabolism: The Metabolic Network of Synechocystis sp. PCC 6803
Source: PLoS Comput Biol. 2013 Jun 27;9(6):e1003081. doi: 10.1371/journal.pcbi.1003081 (PMC3699288; doi:10.1371/journal.pcbi.1003081)
Supplement: Text S2 — Summary of existing reconstructions. (PDF) [file pcbi.1003081.s013.pdf]

An overview of previous reconstructions of Synechocystis sp. PCC 6803

Publication	Year	GRR	#Genes	#Reactions	#Metabolites	GS	Comments
**Shastri and Morgan [10]**	2005	n	--	93	46	y	The first reconstruction. Forward and reverse reactions are treated separately. TheTCA cycle is closed by the glyoxylate shunt.
**Hong and Lee [11]**	2007	y	78	56	54	y	An extension of the model of Shastri and Morgan, inlcuding gene-reaction relationship
**Fu [12]**	2009	y	633	831	704	y	The first genome-scale reconstruction. Only little manual curation.
**Knoop et al. [13]**	2010	y	337	380	291	n	Contains a more detailed representation of photorespiration, the TCA cycle is closed by the GABA shunt.
**Montagud et al. [14]**	2010	y	669	882	790	y	Based on BioCyc software.
**Montagud et al. [15]**	2011	y	811	956	911	y	An extension of the earlier model. The reconstruction in not provided as supplemental information of the article.
**Yoshikawa et al. [16]**	2011	y	376	493	465	y	The study also compares the reconstruction of Knoop et al. and Montagud et al.
**Nogales et al. [17]**	2012	y	678	863	795	n	Makes use of a refined biomass objective function. The TCA cycle is closed by the GABA shunt.
**Saha et al. [18]**	2012	y	731	1156	996	n	Contains the TCA bypass identified by Zhang and Bryant.
**This study**	2013	y	677	759/1035	601	n	Contains the TCA bypass identified by Zhang and Bryant.

GPR: Gene-reaction relationships
GS: Glyoxylate shunt

Reaction and metabolite numbers are taken from the respective network description. Metabolite numbers also contain external metabolites that are not balanced.
